# Evolutionary Genetic Signatures of Selection on Bone-Related Variation within Human and Chimpanzee Populations

**DOI:** 10.3390/genes13020183

**Published:** 2022-01-21

**Authors:** Daryn A. Stover, Genevieve Housman, Anne C. Stone, Michael S. Rosenberg, Brian C. Verrelli

**Affiliations:** 1 School of Life Sciences, Arizona State University, Tempe, AZ 85287, USA; dastover@asu.edu; 2 Arizona State University at Lake Havasu, Lake Havasu, AZ 86403, USA; 3 Section of Genetic Medicine, University of Chicago, Chicago, IL 60637, USA; ghousman@uchicago.edu; 4 School of Human Evolution and Social Change, Arizona State University, Tempe, AZ 85287, USA; acstone@asu.edu; 5 Center for Biological Data Science, Virginia Commonwealth University, Richmond, VA 23284, USA; msrosenberg@vcu.edu

**Keywords:** type I collagen, *COL1A1*, bone disease, BMD, osteoporosis, exon duplication, adaptation

## Abstract

Bone strength and the incidence and severity of skeletal disorders vary significantly among human populations, due in part to underlying genetic differentiation. While clinical models predict that this variation is largely deleterious, natural population variation unrelated to disease can go unnoticed, altering our perception of how natural selection has shaped bone morphologies over deep and recent time periods. Here, we conduct the first comparative population-based genetic analysis of the main bone structural protein gene, collagen type I α 1 (*COL1A1*), in clinical and 1000 Genomes Project datasets in humans, and in natural populations of chimpanzees. Contrary to predictions from clinical studies, we reveal abundant *COL1A1* amino acid variation, predicted to have little association with disease in the natural population. We also find signatures of positive selection associated with intron haplotype structure, linkage disequilibrium, and population differentiation in regions of known gene expression regulation in humans and chimpanzees. These results recall how recent and deep evolutionary regimes can be linked, in that bone morphology differences that developed among vertebrates over 450 million years of evolution are the result of positive selection on subtle type I collagen functional variation segregating within populations over time.

## 1. Introduction

Bone-related disorders impact more than 200 million people globally [1,2,3]. In addition to geographic variation in disorders such as osteoporosis, bone strength in general—measured as bone mineral density (BMD)—also varies among populations, with individuals of African ancestry having better overall bone quality [4,5]. Although variation in bone strength is due in part to environmental differences [6,7], BMD is estimated to be as much as 85% heritable [8,9,10], with hundreds of loci and tens of thousands of variants identified [11,12,13,14]. While our understanding of bone-related variation linked to osteoporotic-related disorders and fractures is incredibly robust due to high-powered GWAS in case–control cohorts [15,16], we still have a limited understanding of natural population variation unrelated to disease, and what evolutionary significance it may have [17].

We expect that bone-related phenotypes are subject to strong purifying selection, yet variation linked to bone loss is not only common, but has likely been segregating in the population for the past 10 Ky [18], with variation attributed to ethnicity, age, and sex [19,20,21]. In fact, the observation of alleles related to increased BMD being significantly more common in sub-Saharan African populations has been attributed to positive selection driving ethnic differences in bone strength [22]. From a deeper evolutionary perspective, phenotypic data for non-human primates, though limited, suggest that genetic variation linked to bone strength also varies within other species [23,24,25]. Chimpanzees, our closest-living relatives with whom we share a common ancestor ~5 Mya [26], show patterns of bone loss and microfractures with age [27,28,29]. With thousands of variants linked to BMD, a top priority is to link functional population variation to therapeutic treatment [16,30]. As such, an overlooked approach is the use of evolutionary genetics to test natural populations for signatures of adaptive functional variation in bone-related genes.

As an evolutionary genetic model, we focused on the collagen type I α 1 (*COL1A1*) gene, which encodes the most abundant protein in mammals and is the main structural protein of bone, teeth, and tendons [31]. *COL1A1* alone possesses more than 600 skeletal and connective tissue disease-associated mutations (DAMs), primarily linked to osteoporosis, osteogenesis imperfecta types I–IV, and Ehlers–Danlos syndromes [32,33]. *COL1A1* is also among the candidate genes commonly linked to natural phenotypic variation in bone strength across ethnic groups [34,35,36]. These factors make this locus a prime candidate for investigating evolutionary factors that shape bone-related phenotypic variation within and among populations and species over time [37].

From the *COL1A1* DAMs previously identified to date, we might conclude that amino acid polymorphism is rare in frequency and associated with disease [32,38]. One bias, however, is that these characterizations come from screenings of clinical individuals with known bone-related disorders. For example, the triple-helix domain of type I collagen is comprised of two COL1A1 and one COL1A2 subunits that wind together, with protein length mutations of any size predicted to have deleterious effects on helix stability [39,40,41]. However, type I collagen genes likely originated hundreds of millions of years ago through a series of duplication events from an ancestral collagen with a single 54 bp exon [42], confirming that variation in length may lead to innovative function [43]. Secondly, the majority of previously identified *COL1A1* DAMs are found in the triple-helix domain, which is a repeating amino acid sequence with glycine in every third position, implying that change to this compact winding structure is deleterious [44,45,46]. However, our previous evolutionary analysis of *COL1A1* sequences from vertebrates spanning ~450 My of divergence showed that the C-terminal domain is more evolutionarily conserved than the triple-helix domain, and the latter exhibits spatial heterogeneity in selective constraint that distinguishes severe from osteoporotic-like phenotypes [37]. Thus, while clinical studies reveal severe *COL1A1* DAMs, evolutionary models show that *COL1A1* variation is historically consistent with both positive selection and disease [47], and predict that natural populations may harbor variation that has functional and adaptive potential.

An additional observation from our previous comparative vertebrate analysis of *COL1A1* was that intron structure and content may be evolutionarily conserved [37], consistent with the need for high gene expression given the importance of type I collagen in vertebrate development and wound repair [48,49]. Indeed, functional studies have shown that sites in the *COL1A1* promoter and first intron regulate gene expression [34,35,50], yet we know little about the evolutionary significance of variation across the > 11 kb of 50 introns. One example comes from a single nucleotide polymorphism (SNP) in an Sp1 transcription factor binding site in the first intron that increases *COL1A1* expression and, likely, accounts for reduced structural integrity and BMD [51,52]. However, this SNP reaches frequencies > 20% in individuals of Western European ancestry, and has also been linked to reduced soft tissue damage [53], suggesting that selective forces are complex. Our previous evolutionary analysis did not examine rate variation in introns, because our approach was limited by a protein-codon-based model [37]. However, in the 10 years since, the field has seen technological and statistical advances, resulting in hundreds of vertebrate genome sequences and evolutionary conservation scores that can be applied to any non-coding nucleotide site [54].

Only one previous study of *COL1A1* in a natural population exists, but with a focus on exons in admixed Americans [55], it does not reveal information on non-coding regions, nor does it reflect varying selective pressures among globally diverse populations. Here, we conduct the first comparative population genetic analyses of *COL1A1* in natural populations of humans and chimpanzees to address the following questions: (1) Is *COL1A1* amino acid polymorphism in natural populations reflective of clinical samples, in that it is highly deleterious and rare or, alternatively, is cryptic protein polymorphism common, as predicted by deep-time evolutionary analyses? (2) Does *COL1A1* intron nucleotide diversity in natural populations reflect high functional constraint or, alternatively, do we find signatures of adaptive evolution suggesting positive selection for gene expression variation? From a larger perspective, comparative population and species analyses help us to understand how deep-time and recent evolutionary pressures are linked and shape adaptive phenotypes vs. disease-related phenotypes across human populations.

## 2. Materials and Methods

### 2.1. Human Population Datasets

We accessed VCFs from the 1000 Genomes Project (www.internationalgenome.org (accessed on 10 December 2021)) [56] for our natural human population sample (Table S1). These data include 2504 individuals (i.e., 5008 *COL1A1* allele copies) with no known bone abnormalities (i.e., a random sample with respect to phenotypic diversity), representing 26 geographically and ethnically diverse populations (“1000G”, hereafter). This sample reflects human genetic diversity within and outside of sub-Saharan Africa, the latter typically having higher nucleotide and haplotype diversity owing to its older, larger, and ancestral effective population size compared to the more recent demographic history of expansion associated with non-African groups [57,58,59,60].

The Leiden Open Variation Database [38] (previously the Osteogenesis Imperfecta and Ehlers–Danlos Syndrome Variant Databases) [32] was used to access hundreds of clinically relevant *COL1A1* disease-associated mutations (“DAMs”, hereafter). The majority of *COL1A1* mutations are associated with osteogenesis imperfecta (OI), and are categorized according to clinical severity of disease symptoms [61]. Similar to our previous analysis [37], we filtered these OI mutations into severity categories of 1–4 (Table S2), from “category 1” OI—reflecting mild bone weakening similar to that caused by osteoporosis, and considered the least severe—to “category 4” OI, reflecting lethality, as the most severe.

### 2.2. Non-Human Primate Population Datasets

We accessed chimpanzee and bonobo raw data and VCFs from the publicly available population genomics project of the *Pan* genus (“*Pan* genomes”, hereafter) [62,63]. This dataset includes individuals from the central (*Pan troglodytes troglodytes*), eastern (*P. t. schweinfurthii*), western (*P. t. verus*), and Nigeria–Cameroon (*P. t. ellioti*) chimpanzee and bonobo (*P. paniscus*) groups. Chimpanzees and bonobos have an estimated divergence time of ~1.6–2.1 Mya, with chimpanzee subspecies groups splitting ~139–633 Kya; however, a complex evolutionary history of gene flow and admixture has been found among groups [63]. Central chimpanzees show the highest nucleotide diversity and lowest long-range linkage disequilibrium (LD), owing to a potentially larger historical population size, whereas the western chimpanzee has consistently shown the lowest nucleotide diversity, no evidence of population structure, and greater separation from other subspecies [62,63,64,65].

From the *Pan* genomes dataset, we sampled 59 individuals from the 4 chimpanzee subspecies (Table S3). This sample comes after multiple filters were applied to the data, removing individuals and nucleotide sites that exhibited unusual patterns (i.e., deviations from Hardy–Weinberg equilibrium). The western subspecies represents the most appropriate contrast with humans, because of similar levels of nuclear population genetic diversity [66,67,68,69,70]. As such, we generated high-coverage nucleotide sequence data from our samples of 20 wild-born, unrelated western chimpanzees (40 allele copies) to complement the *Pan* genomes data. These data include the *COL1A1* locus (Table S4), as well as 20 targeted intergenic regions totaling > 30 Kb spread over 100 Kb in each of the upstream and downstream directions of *COL1A1* (Table S5) to examine long-range LD patterns.

We also sampled bonobo data from the *Pan* genomes data to align with the four chimpanzee subspecies from the same project. We generated high-coverage nucleotide sequence data from 13 wild-born, unrelated bonobos (26 allele copies), to provide alignment for the homologous chimpanzee targeted intergenic regions upstream and downstream of *COL1A1* noted above (Table S6). All western chimpanzee and bonobo sequence data generation followed our previous protocol for targeted PCR, sequencing, and short-read assembly required with the complex repetitive nature of *COL1A1* [37]. Finally, we also used bonobo, orangutan (*Pongo abelii*), and macaque (*Macaca mulatta*) alignments from the UCSC Genome Browser (www.genome.ucsc.edu) [71] to make inferences about derived versus ancestral states of polymorphisms within human and chimpanzee lineages.

### 2.3. Statistical Analyses of COL1A1 Amino Acid Variation

For estimates of evolutionary conservation, we used the vertebrate track for phyloP scores in the UCSC Genome Browser (hg19 assembly), as type I collagen is one of the most abundantly expressed proteins found across all vertebrates. The phyloP statistic identifies rate variation as acceleration (faster) and conservation (slower), and assigns negative and positive scores, respectively, compared to neutrality [54]. The absolute values of the scores represent −log *p*-values under a null hypothesis of neutral evolution, i.e., the closer the value to “0”, the more the evolutionary history of that site conforms to neutral expectations. The vertebrate track shows phyloP evolutionary conservation scores for individual nucleotides for 100 vertebrates, including 62 mammals and 12 primates.

For the DAMs dataset, we investigated the genomic position (hg19 assembly), amino acid change, COL1A1 protein domain, and phyloP score for each single nucleotide mutation (no length or splice variants) (Table S2). The same information was collected from the 1000G dataset, with additional data on the frequencies of the amino acid replacement SNPs within populations (Table S7). We also obtained the same information on amino acid variation within *COL1A1* for the *Pan* genomes dataset, as well as for our generated *Pan* sequence data. Finally, the “severity category” score was determined for each DAM (Table S2), following our previous criteria noted above [37].

We compared distributions of the data categories within and between the DAM and 1000G datasets, i.e., whether SNPs occurred at nucleotide sites with different phyloP scores with respect to severity category, domain, and amino acid change. These distributions were compared using nonparametric statistical tests evaluated for significance via permutations in the coin v. 1.4-2 R package [72].

Our previous evolutionary analyses of *COL1A1* investigated patterns of amino acid divergence across species [37], whereas here we contrast human and chimpanzee amino acid polymorphism. As levels of polymorphism and divergence are expected to be correlated under neutrality, we compared these two classes of variation at *COL1A1* amino acid replacement sites with *COL1A1* intronic sites as proxies for neutrality, using a 2 × 2 test of independence first applied by McDonald and Kreitman [73].

### 2.4. Statistical Analyses of COL1A1 Intronic Variation

As phyloP scores can be calculated for any nucleotide site, the vertebrate track for individual sites was also downloaded (similar to exons above) for the 50 introns of *COL1A1* to determine whether introns and sites underlying SNPs showed unusual evolutionary conservation. Similar to statistical analyses with the coding sequences above, distributions of phyloP scores were compared across datasets using nonparametric statistical tests and permutations to evaluate significance.

We conducted several population genetic analyses to investigate patterns of intronic variation within and between populations and species. First, population-specific estimates of nucleotide diversity for *COL1A1* introns were calculated as Watterson’s [74] *θ_S_*. Second, we used *F_ST_* analyses to investigate patterns of genetic differentiation among human populations and among chimpanzee subspecies. Calculations were conducted using an R script based on the *F_ST_* estimator from Hudson et al. [75] averaged across SNPs between population pairs and, as described by Hudson [76], we used a permutation analysis to identify significant outliers. This analysis pooled populations and randomly sampled allelic variation using our same sample sizes to reconstitute the populations, after which pairwise *F_ST_* values were calculated. This simulation was repeated 1000 times, with observed *F_ST_* values compared to the simulated distributions. These analyses were conducted for the *COL1A1* locus and for SNPs up to 100 Kb upstream and downstream, using the 1000G data for humans and the *Pan* genomes data for chimpanzees.

Finally, to investigate patterns of haplotype structure, we also conducted population-specific LD analyses. For humans, we used the 1000G phased data, and SNP pairwise *r*^2^ values were downloaded using VCFTOOLS v. 0.1.14 [77], after filtering out SNPs with minor allele frequency (MAF) <5%. Population-specific LD data were generated for the *COL1A1* locus, as well as for SNPs up to 100 Kb away in each of the upstream and downstream regions, to investigate long-range haplotype structure. For chimpanzees, we used PHASE v. 2.1.1 [78] to statistically resolve heterozygous sequences for each individual into two haplotypes, after which SNP pairwise *r*^2^ values were generated using DnaSP v. 6.12.03 [79]. The PHASE analysis was repeated with 500, 750, and 1000 iterations with phased haplotypes from the run with the highest average goodness-of-fit used in subsequent analyses. The PHASE and LD analyses were first performed for the *Pan* genomes dataset for each of the four subspecies for *COL1A1*, as well as for SNPs up to 100 Kb away in each of the upstream and downstream regions to investigate long-range haplotype structure. We also performed the same PHASE and LD analyses for our high-coverage western chimpanzee sequence data at *COL1A1*, and for the intergenic regions we collected spanning 100 Kb each upstream and downstream (Tables S4–S6).

## 3. Results

### 3.1. Contrasts of Amino Acid Variation between the DAM and 1000G Datasets

From the 1000G dataset of 5008 global gene copies, we identified 60 amino acid replacement SNPs (Table S7) to compare with the 422 DAMs identified from the clinical database that met the criteria of documented severity categories of 1–4 (Table S2). Each of the 26 populations in the 1000G dataset includes at least one variant, and some include as many as seven (Table S1). Overall, these SNPs are rare in frequency (Table S8); however, we note that 14 of the 60 are found segregating in at least two populations, with one such variant shared across 17 populations and reaching a frequency as high as 7% (Table S7). Interestingly, 6 of these 60 SNPs (10%) occur at the same residues as documented DAMs (Table S9), yet severity can differ across ethnic and age groups [80], especially for “lethal” ones. For example, the Ala-390-Thr variant noted as a category 4 DAM is found segregating in six different populations, five of which are of African ancestry, where we typically expect strength of selection to be higher. However, we note that this variant has a low phyloP score (Table S9), implying a history closer to neutrality, and not strong purifying selection.

We next examined comparisons within and between the DAM categories and 1000G datasets to determine whether evolutionary site conservation distinguishes these groups. First, for some perspective, phyloP scores in the vertebrate track across the genome reach maximum positive values of approximately 10, whereas they can reach maximum negative values of −20 (data unpublished). Even for *COL1A1*’s relatively unusual short exons and introns [37], exon sites have become uncoupled evolutionarily, with significantly higher conservation (reaching the local maximum; Figure S1) compared to introns (Tables S10 and S11). Looking at these sites with respect to DAMs, we find that overall DAMs occur at *COL1A1* sites that are significantly more conserved, but that category 1 DAMs occur at sites with significantly lower conservation compared to categories 2–4 DAMs (Tables S10–S12). Amino acid SNPs in 1000G occur at sites that are significantly less conserved compared to all four DAM categories, and do not occur at *COL1A1* coding sites that are unusually conserved overall (Tables S10–S12). However, amino acid SNPs in 1000G are found at significantly lower frequencies (Table S8; permutation test, *p* = 0.0006), and at sites with significantly higher conservation compared to intron SNPs, which are expected to be putatively neutral in comparison (Tables S10 and S11).

**Figure 1:**
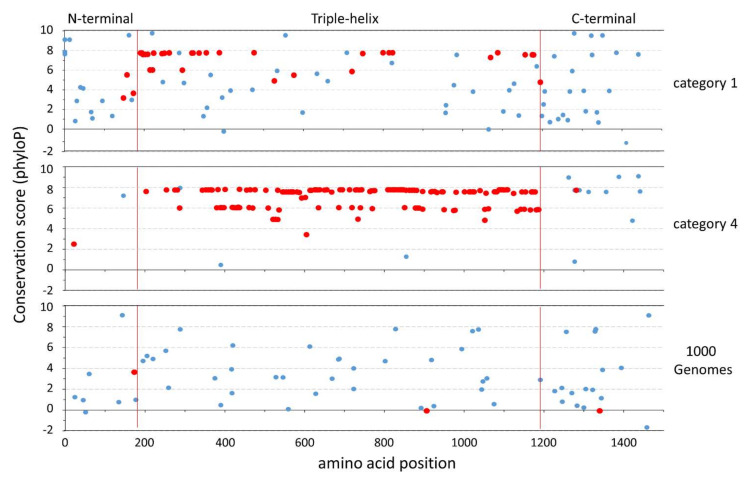
phyloP conservation scores plotted across human COL1A1: phyloP scores are shown for amino acid mutations in disease severity categories 1 (least severe) and 4 (most severe), and in the 1000 Genomes Project dataset across different protein domains (see Table S2). Mutations noted in “red” occur at glycine residues, whereas mutations noted in “blue” occur at non-glycine residues. As phyloP deviates from “0”, the positive and negative scores reflect evolutionary conservation and acceleration, respectively, compared to a neutral model.

We also found that category 2–4 DAMs are more often associated with the triple-helix domain, and least associated with the N- and C-terminal domains, compared to category 1 DAMs (*X*_2_ = 56.9, *p* < 0.001). In addition, category 1 DAMs in the triple helix involve a mutated glycine residue in significantly fewer cases (57%) compared to category 2–4 DAMs (91–98%; Table S13 and Figure S2; Fisher’s exact tests, *p* < 10^−5^). Although the 1000G amino acid SNPs are less associated with the triple helix than category 2–4 DAMs (Table S12, *X*_2_ > 9, *p* < 0.05), they are distributed across domains similar to category 1 DAMs (*X*_2_ = 1.14, *p* = 0.56). That said, the 1000G amino acid SNPs involve a mutated glycine residue in a significantly smaller number of the triple-helix SNPs (2.8%) compared with DAMs (Table S13, Figure 1), even in the least severe category 1 (Fisher’s exact tests, *p* < 10^−7^).

Lastly, McDonald–Kreitman tests found that 1000G amino acid SNPs are unexpectedly common in number (Table S14), especially since there is no amino acid divergence between humans and chimpanzees. Even though this result is typically explained by positive selection for amino acid polymorphism, this pattern may be explained by weak purifying selection on SNPs that remain at low frequencies [81,82]. Indeed, after individually omitting SNPs < 5% in frequency from all classes, there was only one amino acid SNP remaining (in Africans), and all McDonald–Kreitman tests were no longer significant.

### 3.2. Chimpanzee Amino Acid Variation

While we observed no *COL1A1* amino acid divergence between humans and chimpanzees, we also found no amino acid polymorphism within any of the four chimpanzee subspecies in the *Pan* genomes dataset or in our generated sequence data—a total of 158 gene copies. However, we did observe a partial exon 35 duplication that appears to be the result of an unequal crossover event (Figure S3), with an allele frequency of 17.5% (including a homozygous individual), in our high-coverage dataset of 20 western chimpanzees. The exon 35 variant has 36 nucleotides, and the intron splice sites surrounding it are intact; thus, if encoded, it would result in an additional 12 amino acids to the COL1A1 protein in the triple-helix domain.

We further screened additional datasets to determine whether the exon 35 variant was isolated to western chimpanzees. First, the exon 35 variant was not found in the 26 bonobo *COL1A1* copies that we generated. Second, we obtained the raw sequence files for the *Pan* genomes dataset through the European Nucleotide Archive (ENA) Project PRJEB15086 and, through BLAST analysis, identified the exon 35 variant in one western chimpanzee, but not in the other three subspecies. The rare observation of the exon 35 variant in the *Pan* genomes dataset of western chimpanzees is likely the result of the much lower coverage we observed at *COL1A1* compared to the published genome-wide average [63]. This low coverage for *COL1A1* is not surprising, given its highly repetitive exons that are difficult to sequence.

Given the absence of COL1A1 protein coding-length polymorphisms in humans—at least unlinked to severe disease—the observation of one at high frequency in chimpanzees could reveal interesting information about the origin of COL1A1 functional variation. Using PCR analyses, we identified the exon 35 variant in two of the six pluripotent stem cell (iPSC) lines derived from western chimpanzees (Supplementary File S1) [83]. However, our follow-up qPCR analyses of RNA extracted from the two iPSC lines suggest that the exon 35 variant is not incorporated into the *COL1A1* RNA transcript, nor does it appear that it affects *COL1A1* gene expression levels.

### 3.3. Population Patterns of Human Intron Variation

Overall intron nucleotide diversity at *COL1A1* (Table S1) is consistent with the human genome average of ~0.1%, as well as with diversity in Africans being significantly higher than in non-Africans as a result of the aforementioned contrasts in their demographic histories. Previously we noted the overall skew of rare frequencies for amino acid SNPs compared to intron SNPs (Table S8). However, even intron SNP frequencies appeared to be highly skewed, with 87% of them found below 1% in frequency in the global dataset. The majority of this global pattern (88% of SNPs below 1%) is explained by a recent expansion in non-Africans [57]; however, the fact that Africans show very rare frequencies (64% below 1%) suggests that factors other than demography also shape *COL1A1* intron variation overall.

**Figure 2:**
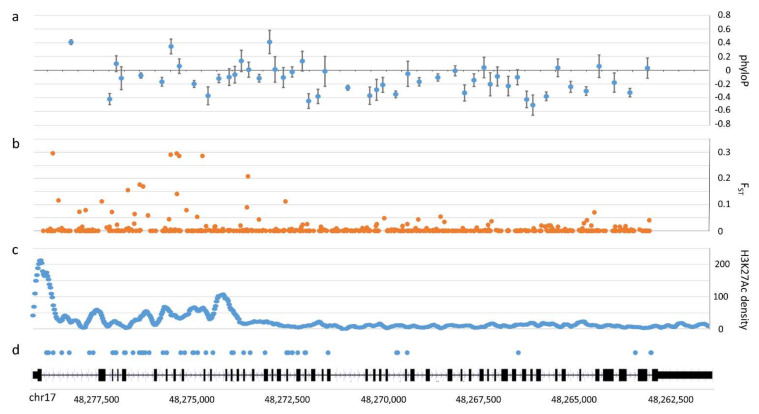
*COL1A1* patterns of variation in humans and chimpanzees: The ~17-kb *COL1A1* locus on chr17 (hg19 assembly) is shown with coding regions as black boxes interspersed with introns. (**a**) phyloP vertebrate track conservation scores for 50 introns (mean and SE plotted at their midpoints). As scores deviate from “0”, the positive and negative scores reflect evolutionary conservation and acceleration, respectively, at nucleotide sites compared to a neutral model. (**b**) Plot of intronic SNP pairwise *F_ST_* values between human populations of African and East Asian ancestry. (**c**) H3K27Ac marks, associated with regulatory elements, with plot reflecting the density calculated as the number of sequenced H3K27Ac tags overlapping a 25 bp window centered at that position. (**d**) Plot of the 45 intron SNPs fixed between the two core haplogroups in 40 western chimpanzee sequences.

In looking at analyses of phyloP score distributions of introns (Tables S10 and S11), 1000G intron SNPs occur at sites with significantly more evolutionary acceleration (−0.36) compared to the distribution observed for all *COL1A1* intron sites (−0.05). However, when we examined the spatial distribution of intron conservation, we found significant heterogeneity (Figure 2a). The first 21 introns that cover a gene distance of ~6700 bp have significantly higher conservation than the latter 29 introns that cover ~8900 bp (randomization test comparing means of introns, while accounting for their lengths, *p* = 0.00085). This pattern was significant even when omitting the first intron (*p* = 0.00057) which, from a genome-wide perspective, is typically longer and more conserved [84].

As is typical with analyses of humans of African descent [85,86], we found significant genetic differentiation for *COL1A1* non-coding SNPs when compared to non-African groups. While the vast majority of SNPs exhibit typical patterns of differentiation among global populations, with *F_ST_* < 15%, there are a few SNPs between Africans and non-Africans that are significant outliers, with *F_ST_* > 30% (Figures S4 and S5; Table S15). As noted previously, there are very few intron SNPs of high frequency, and they are evenly distributed across the *COL1A1* gene (Figure S6). However, SNPs that show significant population structure are located only within introns 1–15 (Figure 2b).

**Figure 3:**
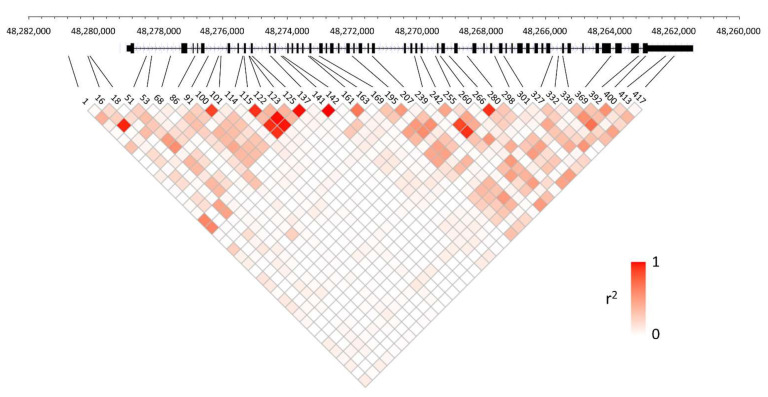
Global pattern of linkage disequilibrium in human *COL1A1*: Linkage disequilibrium pairwise plot of 39 non-coding SNPs (MAF > 5%) in the phased 1000 Genomes Project dataset is plotted as a function of nucleotide distance (see Table S16 for SNP numbering). The ~17 kb *COL1A1* locus on chr17 (hg19 assembly) is shown with coding regions as black boxes interspersed with introns. Shaded boxes in red reflect the strength of pairwise correlations among SNPs.

We found that all *COL1A1* SNPs of high population differentiation (Table S15) are included within a 5′ LD block, which spans the promoter region 2 Kb upstream through intron 15 (Figure 3), and is statistically independent from a 3′ LD block that includes the rest of the gene. We note that these patterns are similar in population-specific analyses (Figure S7), although correlations tend to be stronger and patterns obscured in some non-African populations (Figure S7g–r)—again, as a result of a more recent evolutionary history and less recombination associated with these ancestries [87]. While several intron SNPs are correlated within populations and form smaller blocks, it is clear that correlations among them alone cannot explain the significant population structure. For example, several SNPs that exhibit significant *F_ST_* patterns reach high derived allele frequencies in Africans, or in non-Africans, but are very rare otherwise (Tables S15 and S16). Finally, in looking outside of *COL1A1* (Figure S8), we note that correlations with the 3′ LD block, although weak, continue in the downstream region no further than ~25 Kb, whereas correlations are completely absent between SNPs upstream and SNPs in introns 1–15 that show high *F_ST_*.

### 3.4. Population Patterns of Chimpanzee Intron Variation

Intron nucleotide diversity in *COL1A1* in our sample of 20 western chimpanzees (Table S4) was similar to that of humans, as well as to previously published autosomal loci in these and other samples [66,67,68,69,70]. The phased *Pan* genomes *COL1A1* haplotypes (Table S3) revealed less than one-third of the SNPs in our western chimpanzee dataset (Table S17). This result cannot be explained by the SNPs being rare in frequency (see below), and there is little evidence for population structure in western chimpanzees from the published *Pan* genomes analysis [63]. Instead, this result reflects the significantly reduced sequence coverage of *COL1A1* in the *Pan* genomes dataset. Thus, we used our western chimpanzee sample for standard population genetic analyses, while the *Pan* genomes dataset served for analyses such as LD, where estimates are not biased by missing SNPs.

Unlike the skew in rare allele frequencies for humans, our phased western chimpanzee data revealed two intermediate-frequency haplotypes with strong LD across the *COL1A1* locus (Table S17). In fact, of the 63 intron SNPs in the phased sample, 45 are fixed between the two core haplogroups. In contrast, in our most polymorphic African human sample, with >100 SNPs, only 12 intron SNPs reach an MAF > 30%. Interestingly, 38 of the 45 fixed differences are found in introns 1–21 (Figure 2d)—a result that was significantly unexpected (*p* < 10^−7^) based on a permutation analysis that simulated random distributions of 45 mutations across the >11 Kb of 50 introns. This result shows that the high proportion of fixed differences between haplogroups occurring in the first 21 introns is highly unlikely to be explained by chance alone.

Although far fewer SNPs exist in the *Pan* genomes dataset of *COL1A1*, we can see that the unusual haplotype structure in western chimpanzees is also similar across the other three subspecies (Table S3), suggesting a relatively old age that predates chimpanzee subspecies divergence. To test this hypothesis, we employed the model of Thomson et al. [88], wherein assumptions of population equilibrium and recombination are relaxed. Owing to the unusually high LD, even across subspecies, very few recombinants are obvious, enabling more accurate age estimates under such a coalescent approach [89,90]. The age estimate (*t*) involves the relationship in (1):

(1) $$t=\sum_{i=1}^{n}\frac{x_{i}}{\left(n\mu \right)}$$

where *x_i_* is the number of mutational differences between the *i*th sequence and the most recent common ancestor (MRCA) of all sequences, *n* is the number of sequences, and *µ* is the mutation rate. Here, *µ* is estimated as the number of substitutions between human and chimpanzee divided by the estimated molecular divergence time between species (5 ± 1 My) [26], multiplied by two. Alignments with bonobo and human data enabled estimates of *x_i_* as the number of differences accumulated on each haplotype since the MRCA. Our estimate of the divergence time between the two haplotypes was 2.8 ± 0.6 My, which predates not only chimpanzee subspecies divergence times, but also the chimpanzee–bonobo divergence time [63].

**Figure 4:**
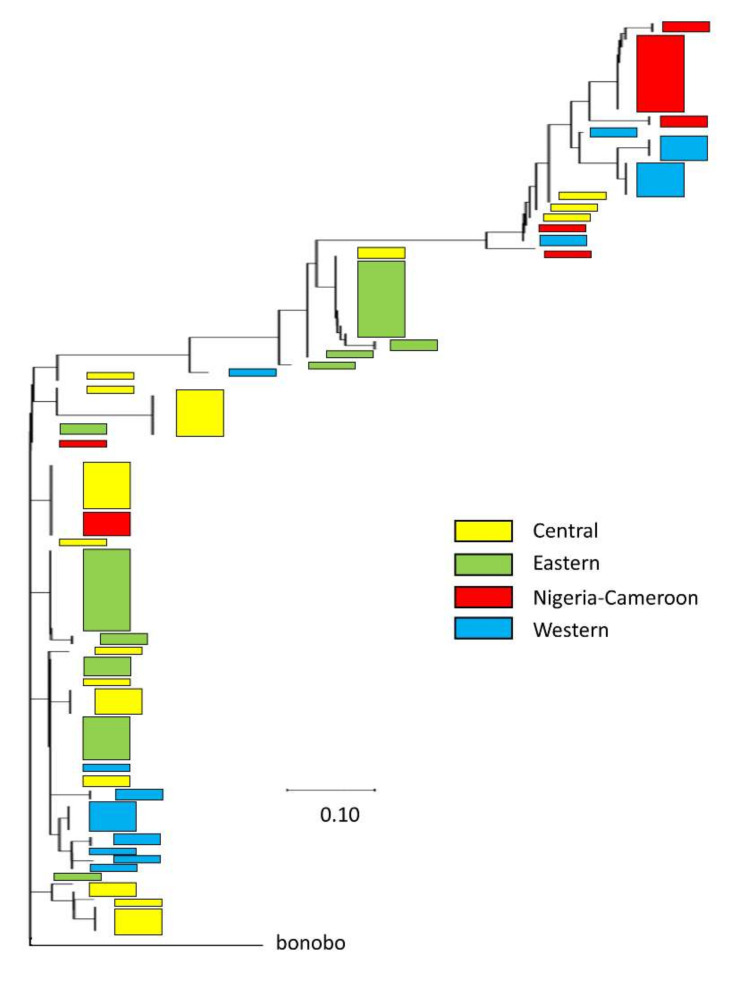
Neighbor-joining tree of 118 chimpanzee *COL1A1* haplotypes: Haplotypes and their relative phylogenetic positions for each of the four subspecies are shown as color blocks, with bonobo as an outgroup. Evolutionary distances reflect the number of base substitutions per variable site.

Inspection of the bonobo data from the *Pan* genomes dataset shows that bonobos are not fixed for either of the two chimpanzee core haplotypes, but are a mix of the two (Table S17). This pattern is consistent with the two haplogroups existing in the ancestral population prior to the divergence of chimpanzee and bonobo, and with our estimated date above. One explanation for the relatively old age, yet such high LD, could be historical introgression of haplotypes from bonobos, as has been found for some chimpanzee subspecies, but very rarely in western chimpanzees. To test this hypothesis, we generated neighbor-joining (NJ) phylogenetic trees using MEGA-X [91] for (1) *COL1A1* in our 40 phased western chimpanzee sequences, with bonobo as an outgroup (Table S17); (2) sequenced regions 100 Kb upstream and downstream in our 40 phased western chimpanzee (Table S5) and 26 phased bonobo sequences (Table S6), using human as an outgroup; and (3) *COL1A1* in the *Pan* genomes’ 118 phased subspecies sequences, using bonobo as an outgroup (Table S3). The western chimpanzee *COL1A1* NJ tree (Figure S9) reflects the two haplogroups seen in our high-coverage data, with divergence (albeit little) between chimpanzees and bonobos. This monophyletic pattern is even more apparent in the NJ tree that includes the upstream and downstream regions (Figure S10). Our final NJ tree analysis shows *COL1A1* haplotypes shared across the four subspecies (Figure 4), but as a monophyletic group separate from bonobos. Thus, we find no evidence of introgression of bonobo alleles into chimpanzees at *COL1A1* or areas in close proximity that can explain the unique *COL1A1* haplotype structure.

Finally, we used long-range LD and estimates of *F_ST_* from the *Pan* genomes dataset to test the hypothesis that the haplotype structure of *COL1A1* is the result of linkage with other loci, or even potentially the result of hybridization among chimpanzees. Calculations of LD between a core set of SNPs at *COL1A1* and other SNPs in each of the four subspecies samples show a striking pattern of localized LD at *COL1A1* that variably decays, moving 100 Kb each upstream and downstream (Figure S11). This decay is expected, as recombination reduces correlations as a function of time and population size and, as predicted, is most abrupt in central chimpanzees, but with the least decay in Nigeria–Cameroon samples [63,92]. This same pattern can be visualized via the *F_ST_* analyses that, despite the high sharing of haplotype diversity in *COL1A1* among subspecies, show high levels of differentiation between subspecies over a 1 Mb region outside of *COL1A1* (Figure S12). These results demonstrate that the old chimpanzee haplotype structure localized to the *COL1A1* locus cannot be explained simply by demographic or chromosomal factors.

## 4. Discussion

The present study takes advantage of phylogenetic conservation analyses, whole-genome databases, and molecular functional tools that have emerged in the 10 years since our initial deep-time evolutionary analyses of *COL1A1* suggested that the general population likely harbors significantly more variation than clinical studies reveal [37]. We found contrasting patterns of *COL1A1* amino acid variation between clinical and natural human populations, and multiple evolutionary signatures of potentially adaptive functional variation associated with introns in humans and chimpanzees. Here, we discuss the implications that these observations may have for the selective pressures shaping bone-related phenotypes, and consequences for disease prevalence.

### 4.1. COL1A1 Protein Variation Is Higher Than Expected

COL1A1 protein variation in the natural population appears more common than would be expected if it were simply linked to severe disease. From a broader perspective, this pattern is unusual even in comparison to genome-wide patterns for “disease genes” [90,93,94,95]. One consideration is that studies have shown that sequencing anomalies in the 1000G data impact estimates of rare alleles [96,97]; however, there are several reasons why these anomalies alone cannot explain our results. First, we would expect all populations and sites to be similarly impacted, yet we see that patterns differ between Africans and non-Africans, and between coding and non-coding variants. Second, the patterns of human population differentiation for intronic variation discussed below all involve common variants. Lastly, as we presented high-coverage data in a resequencing of chimpanzees and bonobos, we also conducted a similar resequencing effort of *COL1A1* in a global sample of humans (data unpublished, Table S18), finding that 9% of individuals carry at least one amino acid variant. This result is consistent with the 1000G data—specifically, the global human sample predicts that ~5% of individuals, and as many as 9% of individuals with African ancestry, carry an amino acid variant in *COL1A1*.

Compared to DAMs, patterns for natural populations are consistent with the hypothesis that *COL1A1* variants do not always reflect disease [80] but, rather, they represent a different category of COL1A1 protein variation altogether. In regions of the protein such as the triple helix, where change is expected to be deleterious, they fall almost completely at sites different from DAMs, and have significantly lower conservation scores, even compared to the least severe category of osteoporotic-like disease (Figure 1). In other words, amino acid polymorphism at *COL1A1* appears to have varying functional effects insufficient to result in severe disease and subsequent detection in clinical studies, and may thus contribute to natural phenotypic variation in type I collagen that is not deleterious with respect to fitness. In this respect, patterns of polymorphism and divergence in *COL1A1* are actually consistent with the same evolutionary process. That is, certain mutations are rapidly removed by purifying selection (i.e., fatal mutations impairing the triple-helix domain [32,33]), resulting in little fixation over deep time, while others with subtle to no effect on type I collagen can accumulate as polymorphisms in the general population. As such, purifying selection at *COL1A1* would not be considered “weak”, as has been suggested as an explanation for similar patterns of genome-wide amino acid variation [93,94,95]; rather, selection is of varying strength across the protein sequence over time. This scenario was initially proposed by us and others from deep-time evolutionary analyses [37,47]. Specifically, variation in constraint along the COL1A1 triple-helix domain implies more flexibility in this region over evolutionary time, which makes sense, as this region is responsible for structural and mechanical variation in bone, including mineral content and organization of collagen fibers that vary among vertebrates [98].

These combined deep-time and population-based evolutionary analyses make predictions about where mutations of variable functional impact are likely to occur and contribute to population variation related to bone morphology. For example, only 5% of DAMs are found in the N-domain, with almost all of them related to osteoporotic-like diseases; in contrast, when looking at the natural population, we find that 13% are found in the N-domain. We see a similar pattern for the C-domain, with 15% and 28% found in DAMs and the natural population, respectively. While the N-domain helps keep the protein soluble until translation and processing are complete, the C-domain is responsible for the recognition and assembly of type I collagen subunits, and where mutations can be severe, as they prevent formation of the triple-helix [44,45]. This result may be expected for the C-domain which, as previously mentioned, shows the strongest signature of deep-time evolutionary constraint in our previous analyses [37], supporting the patterns seen here at the population level. Finally, we also identified mutations—even shared across populations—in specific regions of the triple-helix domain that are predicted to be “lethal” because they are related to major ligand binding [33]. Thus, while *COL1A1* mutations have been linked to disease severity, natural populations represent complex interactions between the environment and natural selection that have resulted in some mutations being common. While these patterns cannot explain the overall variation in phenotypic and functional differences, such as BMD, across populations [22], they do represent variation that can be evolutionarily vital to bone-related phenotypes over time. That is, factors such as amino acid size and thermostability are important to bone-related disease prediction models [33,41], whereas subtle variation that does not greatly impact phenotypes has likely acted as adaptive potential for selection in historically shaping type I collagen [42,43].

A great example of this adaptive potential comes from our analyses of chimpanzees. The absence of observed amino acid polymorphism in all four subspecies of chimpanzees, and in bonobos, may reflect different environmental constraints between our species, such as in locomotion, diet, and skeletal growth periods [99,100]. However, the common variant resulting in a partial duplication of exon 35 was an unusual find compared to humans, where mutations that affect protein length—and particularly of the triple-helix domain—are exceedingly rare and highly deleterious [39,40,41]. Unsurprisingly, we found no evidence that the variant is currently encoded, as it would likely require a convergent change in COL1A2 to accurately form type I collagen. However, it should be noted that the current family of collagens all likely evolved from an ancestral collagen with a single 54 bp exon [42]; thus, mutation in length variation must have been available historically across vertebrate evolution. The nature of the exon 35 variant with a truncation of the exon that would code for 12 amino acids—with all splice sites intact, and at high frequency—represents an example of intriguing standing genetic variation of potential functional value for positive selection.

### 4.2. Signatures of Adaptive COL1A1 Intronic Variation within Humans and Chimpanzees

The patterns of deep-time evolutionary constraint, human population differentiation, and unusual chimpanzee haplotype structure all coincidentally isolated to the same intronic region of *COL1A1* cannot be explained by neutral forces such as shared demographic history, population structure, or low recombination. One explanation is that balancing selection—which favors intronic diversity within species, but reduces divergence over time [101,102]—maintains variation in *COL1A1* expression. In looking at ChIP-seq data for the *COL1A1* locus [103], enrichment of H3K27Ac—well recognized as a marker of enhancer activity and gene expression—is significantly over-represented not only in the first intron, but at least through intron 16 (Figure 2c), which coincides with the multiple signatures of selection seen here. The first intron includes numerous transcription-factor-binding sites [34,35,50], and frequencies of the Sp1 allele found here at 20% in Europeans and 9% in Africans confirm that it has long been segregating in the human population. However, the Sp1 allele is not one of the intron variants identified here that show patterns of significant population differentiation. Interestingly, these intronic patterns all include populations with sub-Saharan African ancestry, with intronic SNPs of intermediate derived allele frequencies that are virtually absent elsewhere. A previous study suggested that positive selection explains the over-representation of alleles that increase BMD in sub-Saharan African populations [22]. Thus, we might conclude that the signatures of positive selection associated with *COL1A1* intronic alleles over-represented in our sub-Saharan African sample also reflect increased BMD as a result of variation in *COL1A1* gene expression. In this respect, bone strength joins a list of phenotypes—such as malarial resistance [69,104], color vision [70,105], lactase persistence [106], and lipid and glycemic traits [107]—that have evolved from positive selection for adaptive immunity and subsistence in ancestral sub-Saharan Africans, yet are often linked to disease elsewhere [58,59,90,94]. These trait differences reflect variable selective pressures over time across ethnic groups and species, and merit caution in drawing conclusions about how phenotypic and genetic variation in one environment may have similar deleterious or advantageous effects in another.

## 5. Conclusions and Future Directions

Identifying the relationship between genotype and phenotype is a challenge for which evolutionary genetic studies provide a unique perspective, with additional insight into the adaptive potential of this variation. In this respect, disease phenotypes such as those associated with COL1A1 provide an excellent starting point, as we have intimate information about genotype–phenotype relationships that enables us to interpret natural population variation. Our results here are consistent with evolutionary theory in general, in that the *COL1A1* variation most likely to have adaptive potential would be subtle in nature with respect to phenotype [108], and would most likely go unnoticed in clinical screenings.

We can speculate as to whether the signatures of adaptive collagen-related variation here are the result of historical selection for bone strength related to locomotion, development, and wound repair [99,100]; however, a future step would be first to determine the extent to which these variants represent functional variation. Examples include targeted *COL1A1* SNPs in human- and chimpanzee-derived cell lines with RNA-seq, ChIP-seq, and reporter assay technologies to identify the effects of gene expression [109], as well as investigations of animal models and case–control cohorts that exhibit different BMD profiles [16,110]. As comparative primate functional genomics continues to evolve [111], we predict that using our study of *COL1A1* as a model for other gene-based evolutionary analyses will reveal cryptic variation underlying “disease genes” with potential functional and adaptive significance.

## Data Availability

Not applicable.

## Supplementary Materials

The following are available online at https://www.mdpi.com/article/10.3390/genes13020183/s1, Figure S1: phyloP vertebrate track conservation scores across *COL1A1*, Figure S2: phyloP scores for human COL1A1 amino acid mutations across disease severity categories and protein domains, Figure S3: *COL1A1* exon 35 partial duplication in western chimpanzees, Figure S4: African vs. non–African F_ST_ plots of *COL1A1* intronic SNPs, Figure S5: Non-African F_ST_ plots of *COL1A1* intronic SNPs, Figure S6: Plot of minor allele frequencies for 436 *COL1A1* intron SNPs in the 1000 Genomes dataset, Figure S7: Linkage disequilibrium pairwise SNP plot across the human *COL1A1* locus, Figure S8: Linkage disequilibrium pairwise SNP plot for human chromosome 17 region including *COL1A1*, Figure S9: Neighbor-Joining phylogenetic tree of 40 *COL1A1* haplotypes from western chimpanzees, Figure S10: Neighbor-Joining phylogenetic tree of a 220 Kb region of chromosome 17 centered on *COL1A1* from 40 haplotypes from western chimpanzees and 26 haplotypes from bonobos, Figure S11: Linkage disequilibrium plot across chimpanzee chromosome 17 region including *COL1A1*, Figure S12: Chimpanzee F_ST_ plot across chromosome 17 region including *COL1A1*, Table S1: 1000 Genomes Project population samples and *COL1A1* summary data, Table S2: Clinical database of *COL1A1* DAMs (Disease-Associated Mutations), Table S3: Chimpanzee *COL1A1* haplotypes, Table S4: *COL1A1* nucleotide diversity in 40 western chimpanzee sequences, Table S5: Chimpanzee nucleotide diversity in non-coding regions upstream and downstream of *COL1A1*, Table S6: Bonobo nucleotide diversity in non-coding regions upstream and downstream of *COL1A1*, Table S7: Human *COL1A1* amino acid variants in the 1000 Genomes Project, Table S8: 1000 Genomes Project distributions of *COL1A1* intron SNPs and amino acid variants, Table S9: *COL1A1* amino acid SNPs shared between 1000 Genomes and DAM databases, Table S10: PhyloP score distributions and comparisons for different *COL1A1* datasets, Table S11: Statistical tests of phyloP distributions for *COL1A1* categories, Table S12: Summary data for *COL1A1* amino acid variant datasets, Table S13: Comparisons across human datasets of proportion of triple-helix mutations in *COL1A1* that mutated from a glycine residue, Table S14: McDonald-Kreitman tests of neutrality at human *COL1A1*, Table S15: 1000 Genomes *COL1A1* SNPs with significant population differentiation using Fst, Table S16: 1000 Genomes Project phased *COL1A1* SNPs for LD analyses, Table S17: Western chimpanzee *COL1A1* haplotypes, Table S18: *COL1A1* amino acid variants revealed from re-sequencing in a global sample of 96 humans (192 gene sequences), File S1: Characterization of chimpanzee *COL1A1* exon 35 partial duplication.
