# Supplementary material for: Evolutionary Genetic Signatures of Selection on Bone-Related Variation within Human and Chimpanzee Populations
Source: Genes (Basel). 2022 Jan 21;13(2):183. doi: 10.3390/genes13020183 (PMC8871609; doi:10.3390/genes13020183)

## Slide 1
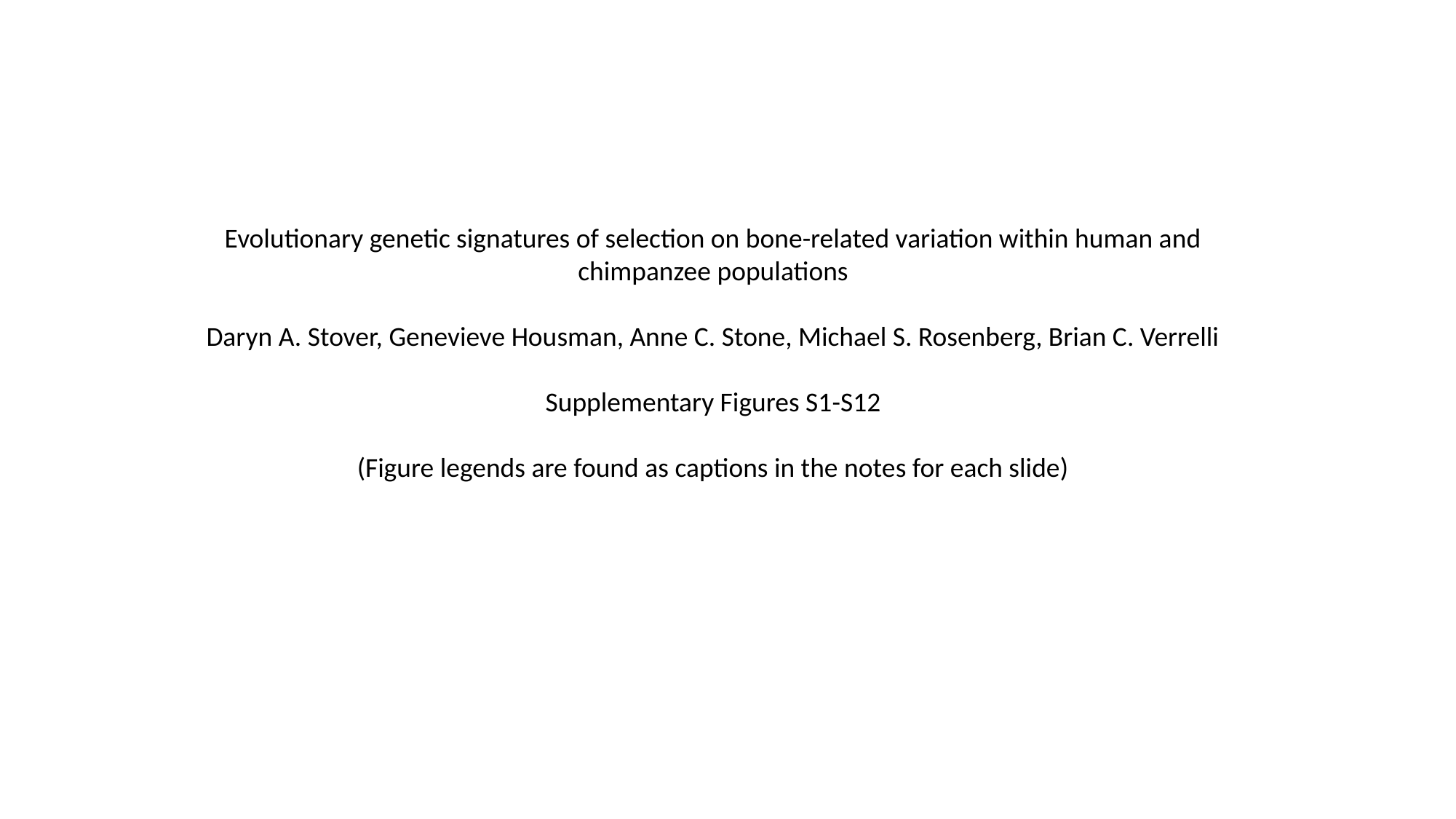

Evolutionary genetic signatures of selection on bone-related variation within human and chimpanzee populations
Daryn A. Stover, Genevieve Housman, Anne C. Stone, Michael S. Rosenberg, Brian C. Verrelli
Supplementary Figures S1-S12
(Figure legends are found as captions in the notes for each slide)

## Slide 2
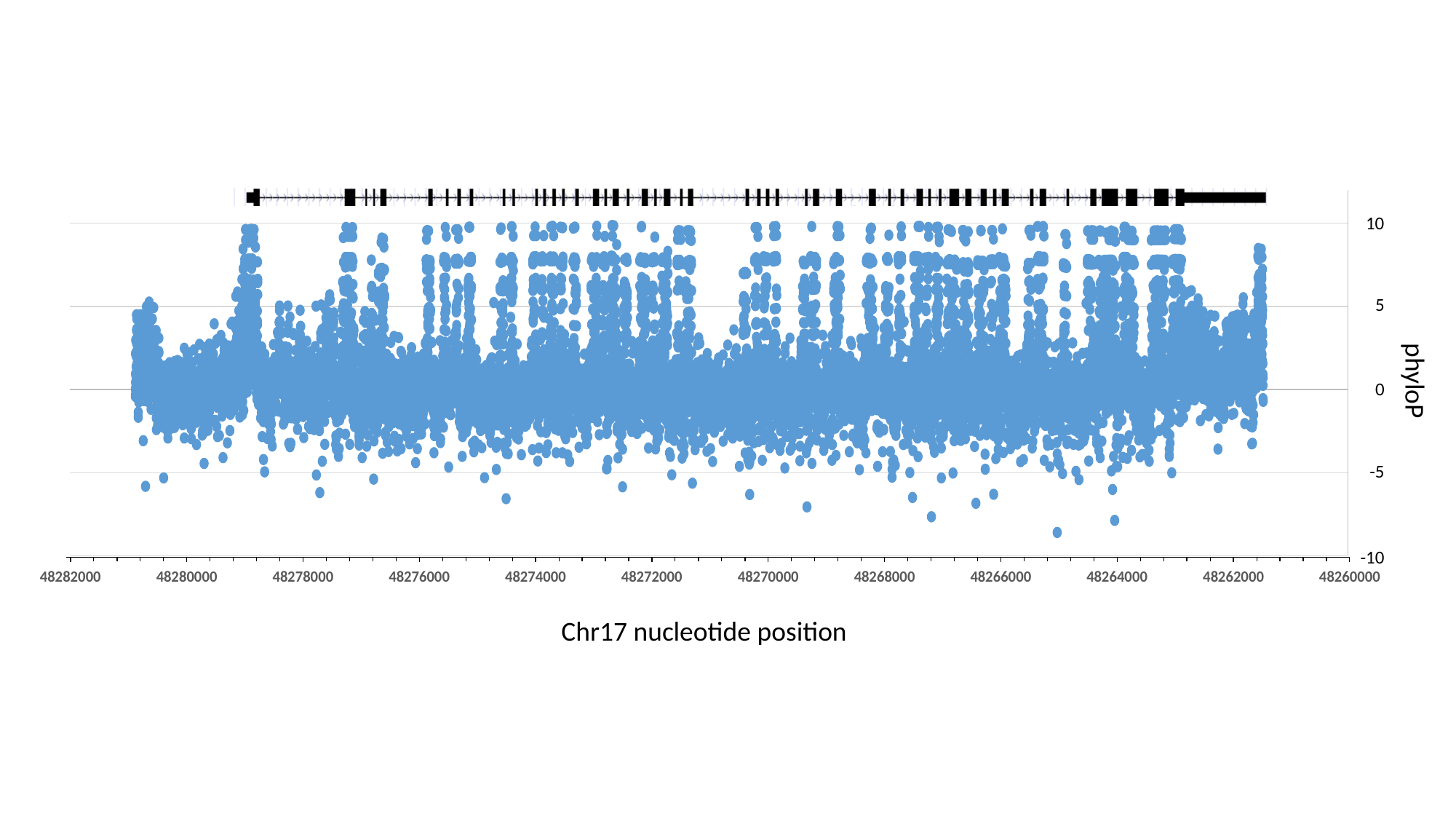

10
5
0
-5
-10
phyloP
Chr17 nucleotide position

## Slide 3
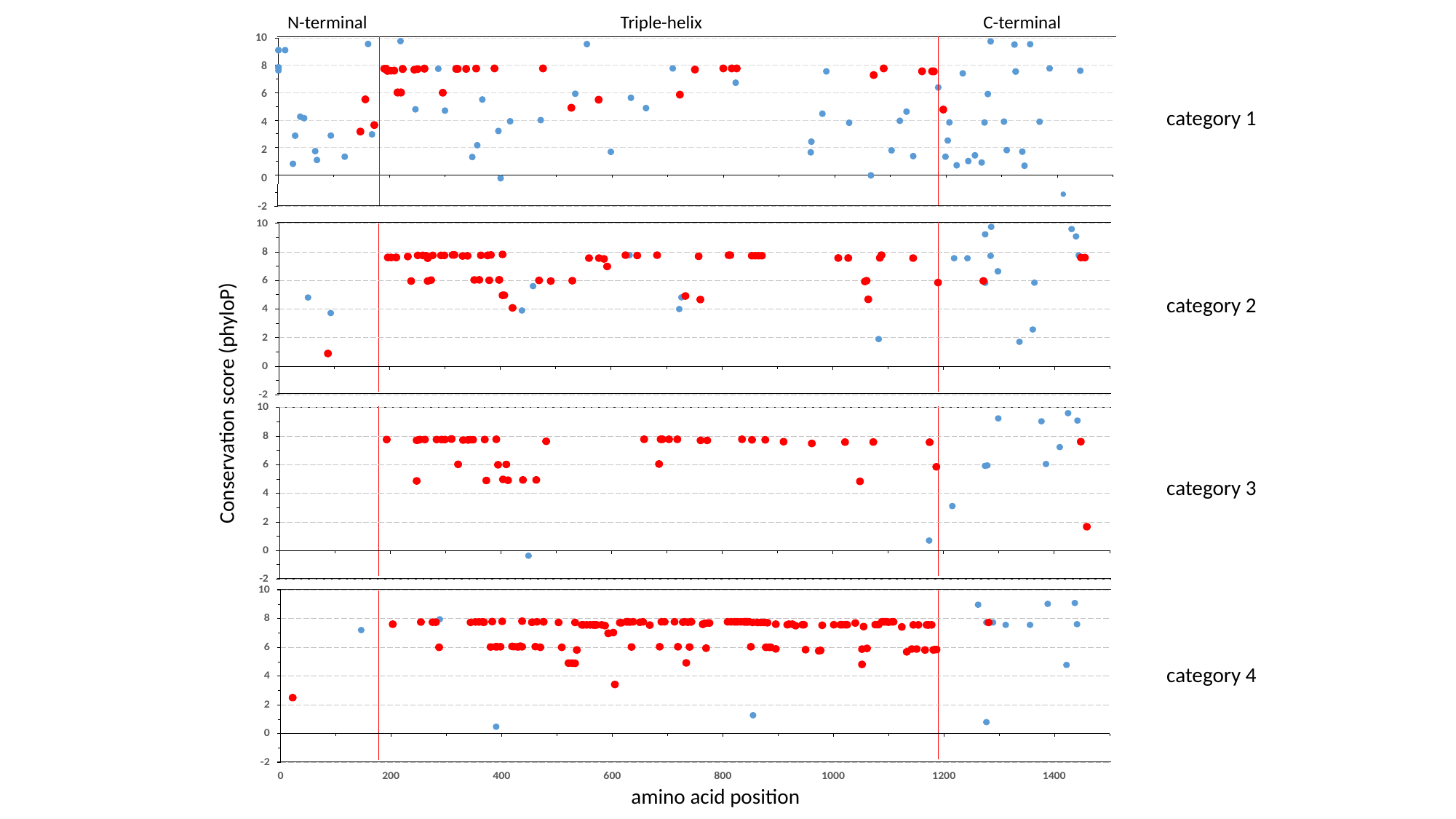

N-terminal
Triple-helix
C-terminal
category 1
category 2
Conservation score (phyloP)
category 3
category 4
amino acid position

## Slide 4
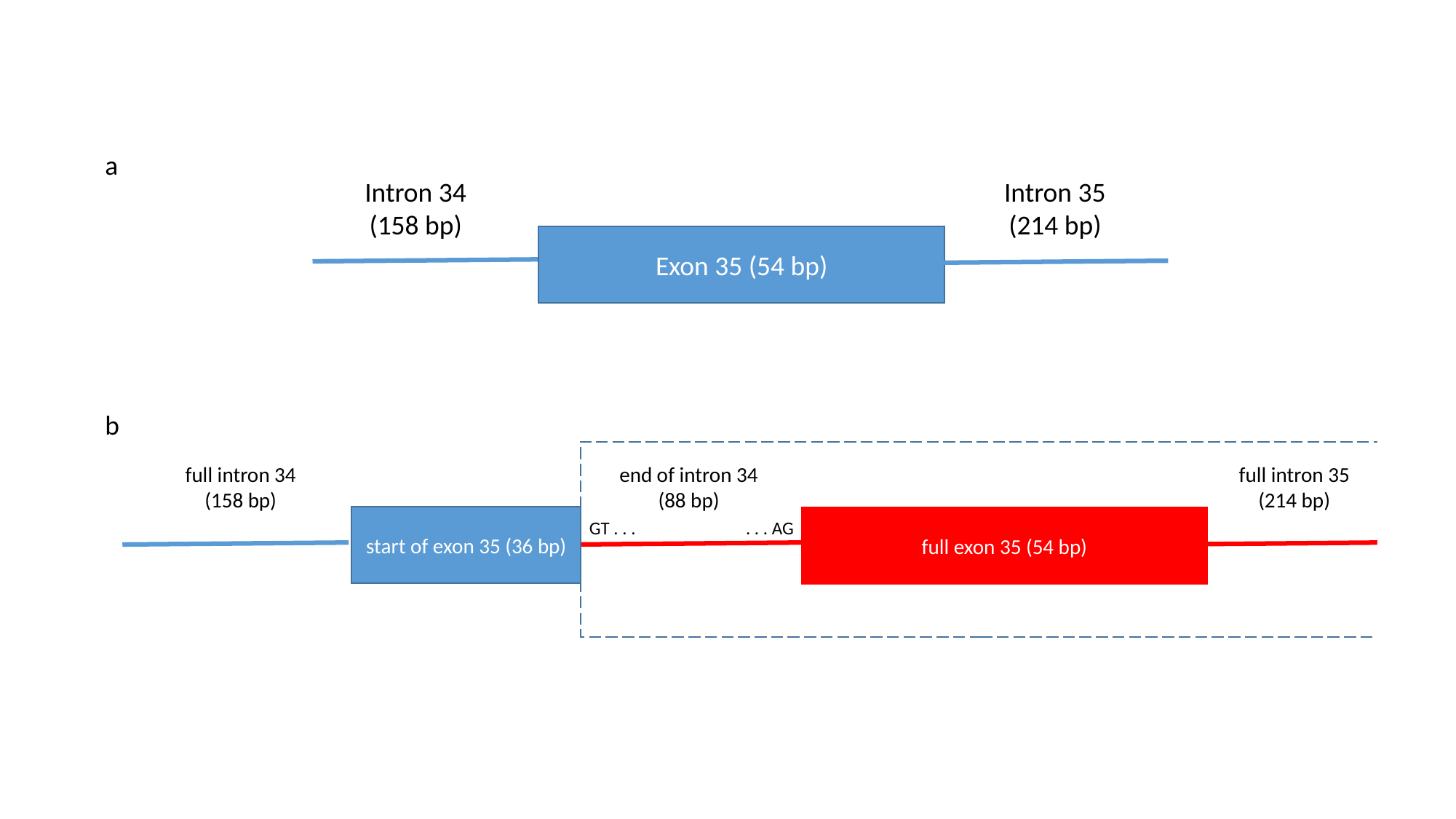

a
Intron 34
(158 bp)
Intron 35
(214 bp)
Exon 35 (54 bp)
b
full intron 34
(158 bp)
end of intron 34
(88 bp)
full intron 35
(214 bp)
start of exon 35 (36 bp)
full exon 35 (54 bp)
GT . . .
 . . . AG

## Slide 5
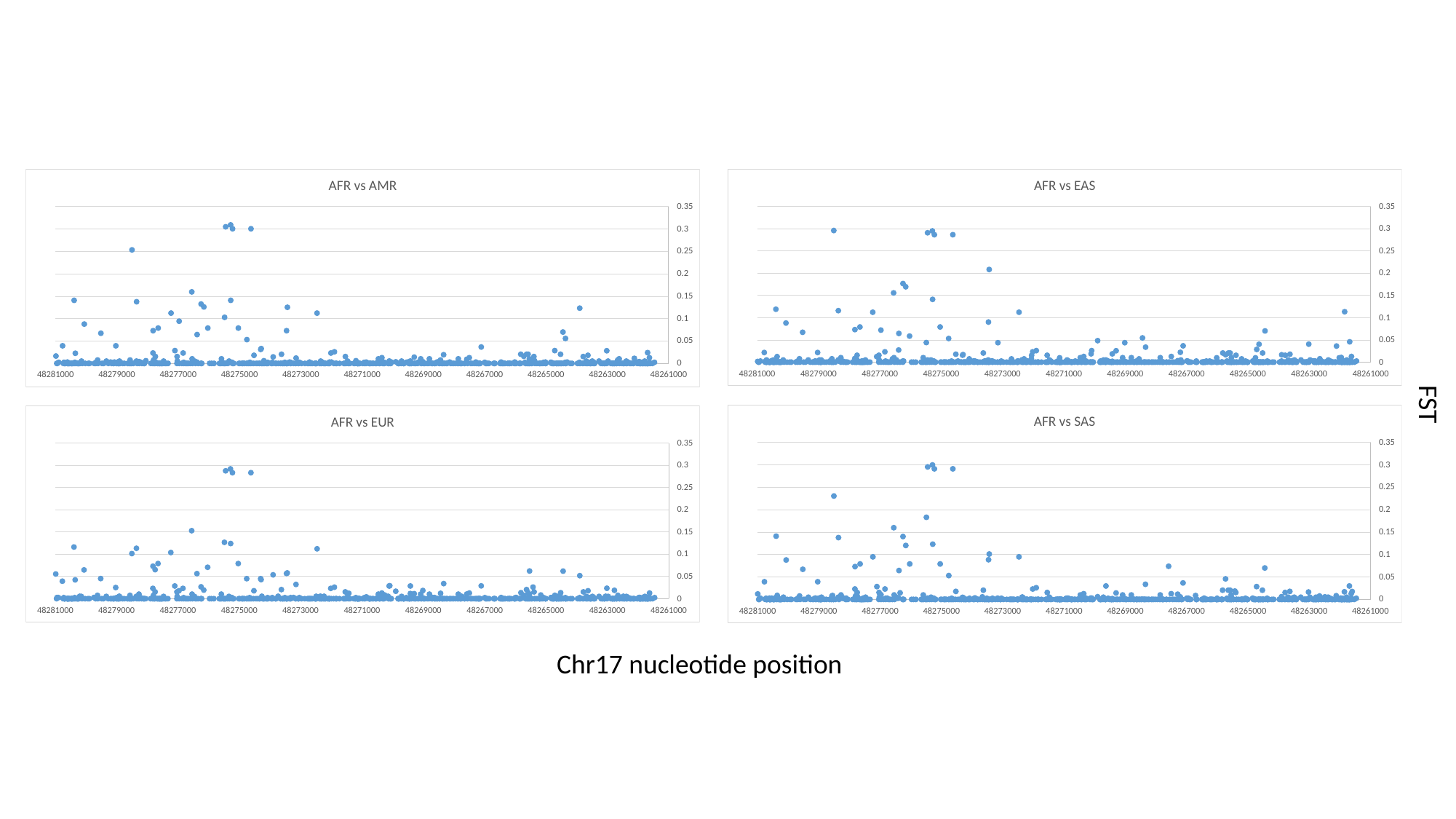

FST
Chr17 nucleotide position

## Slide 6
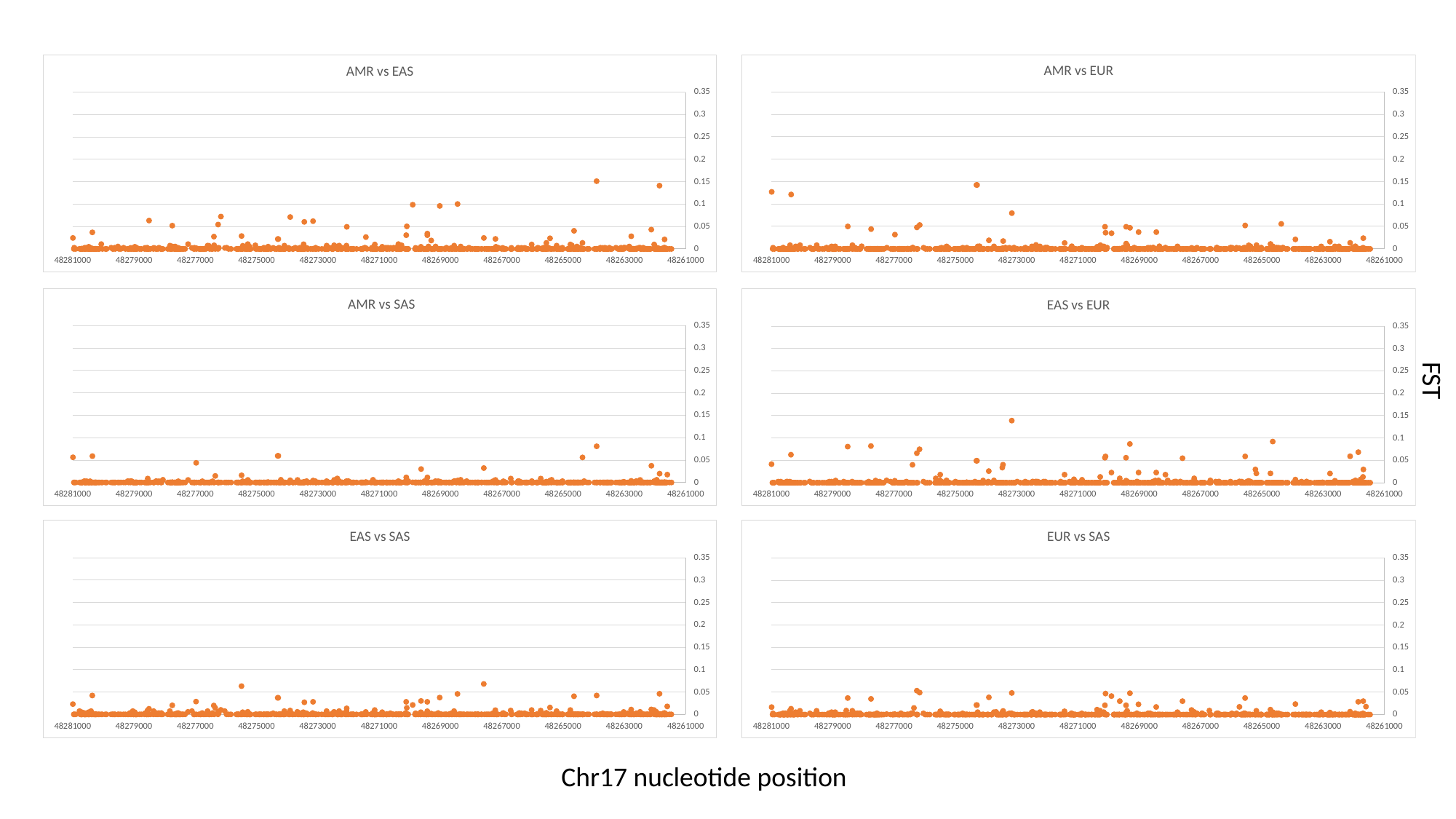

FST
Chr17 nucleotide position

## Slide 7
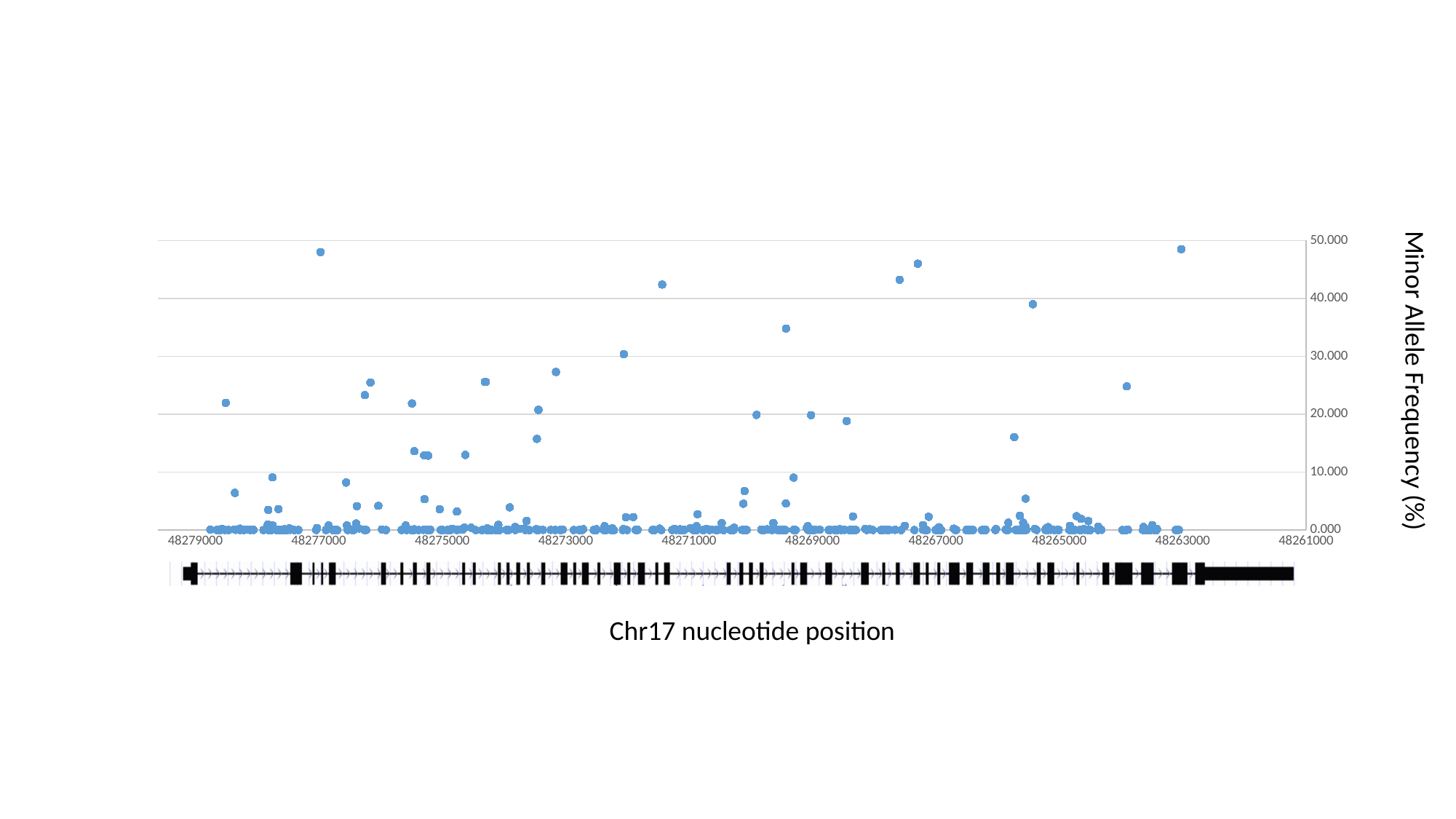

### Chart
| Category | |
|---|---|
Minor Allele Frequency (%)
Chr17 nucleotide position

## Slide 8
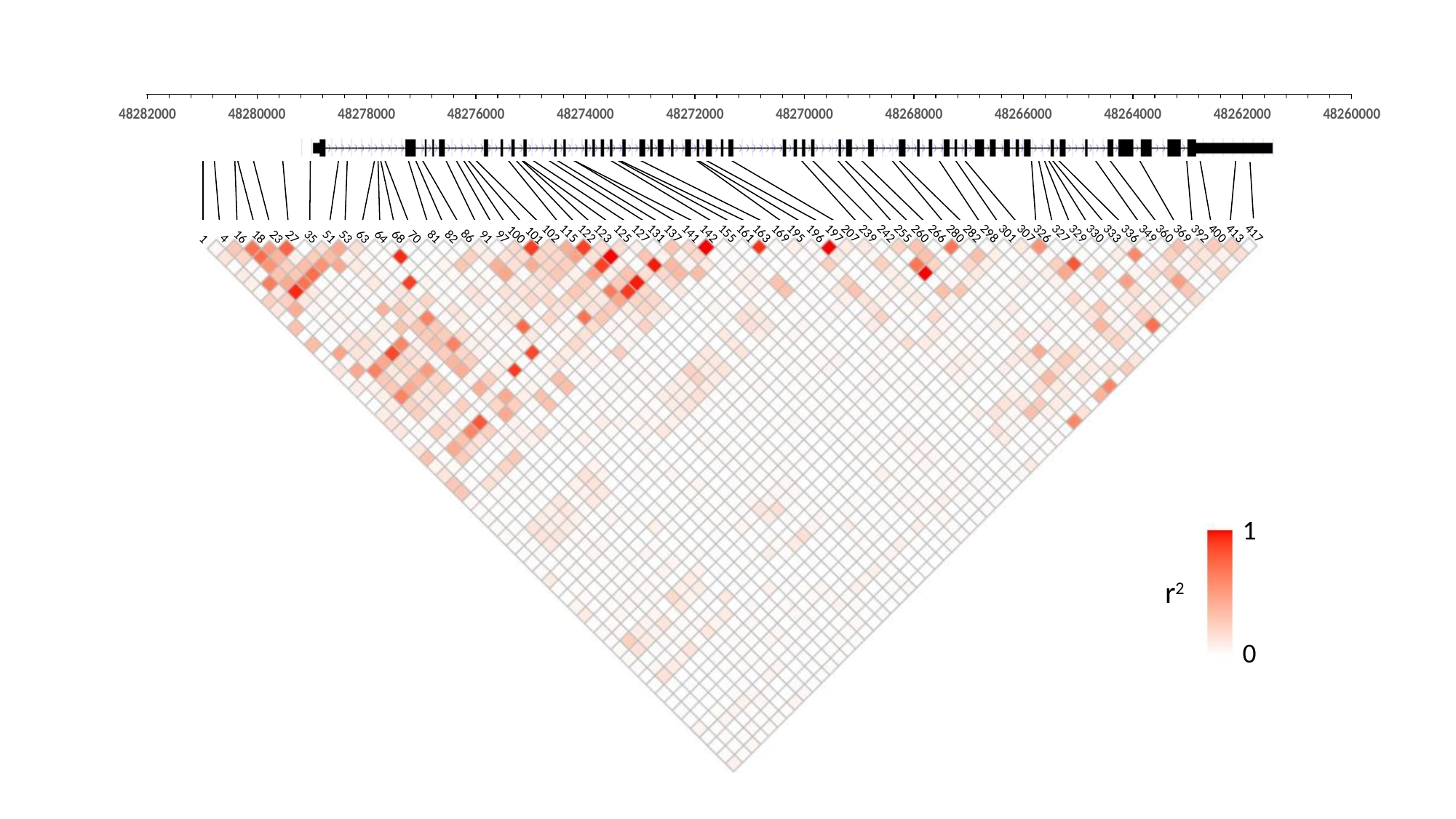

102
115
122
123
125
127
131
137
141
142
155
161
163
169
195
196
197
207
239
242
255
260
266
280
282
298
301
307
326
327
329
330
333
336
349
360
369
392
400
413
417
100
101
16
18
23
27
35
51
53
63
64
68
70
81
82
91
97
 86
4
1
1
r2
0

## Slide 9
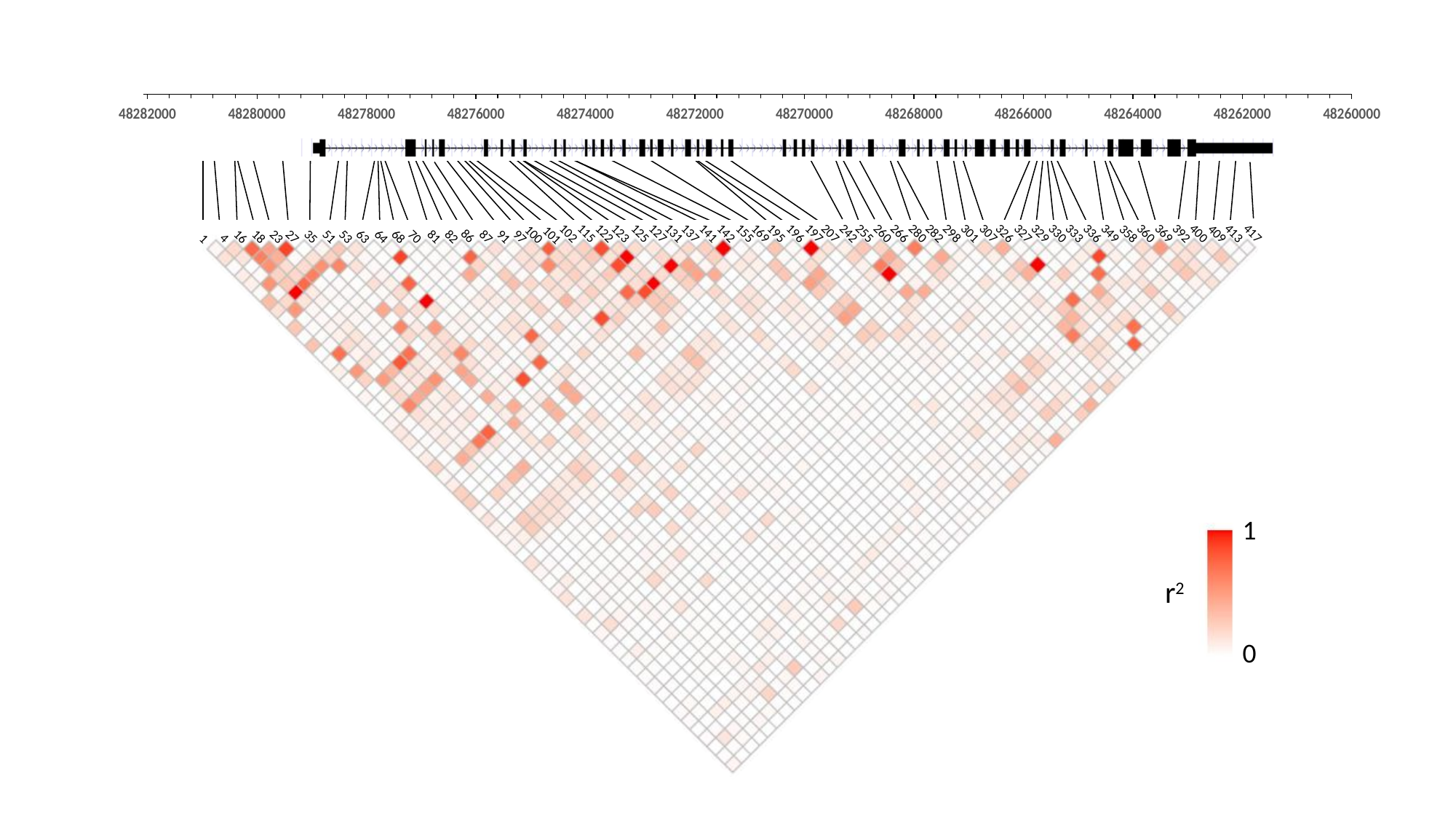

102
115
122
123
125
127
131
137
141
142
155
100
101
97
169
195
196
197
207
242
255
260
266
280
282
298
301
307
326
327
329
330
333
336
349
360
369
392
400
413
417
409
358
16
18
23
27
35
51
53
63
64
68
70
81
82
87
 86
91
4
1
1
r2
0

## Slide 10
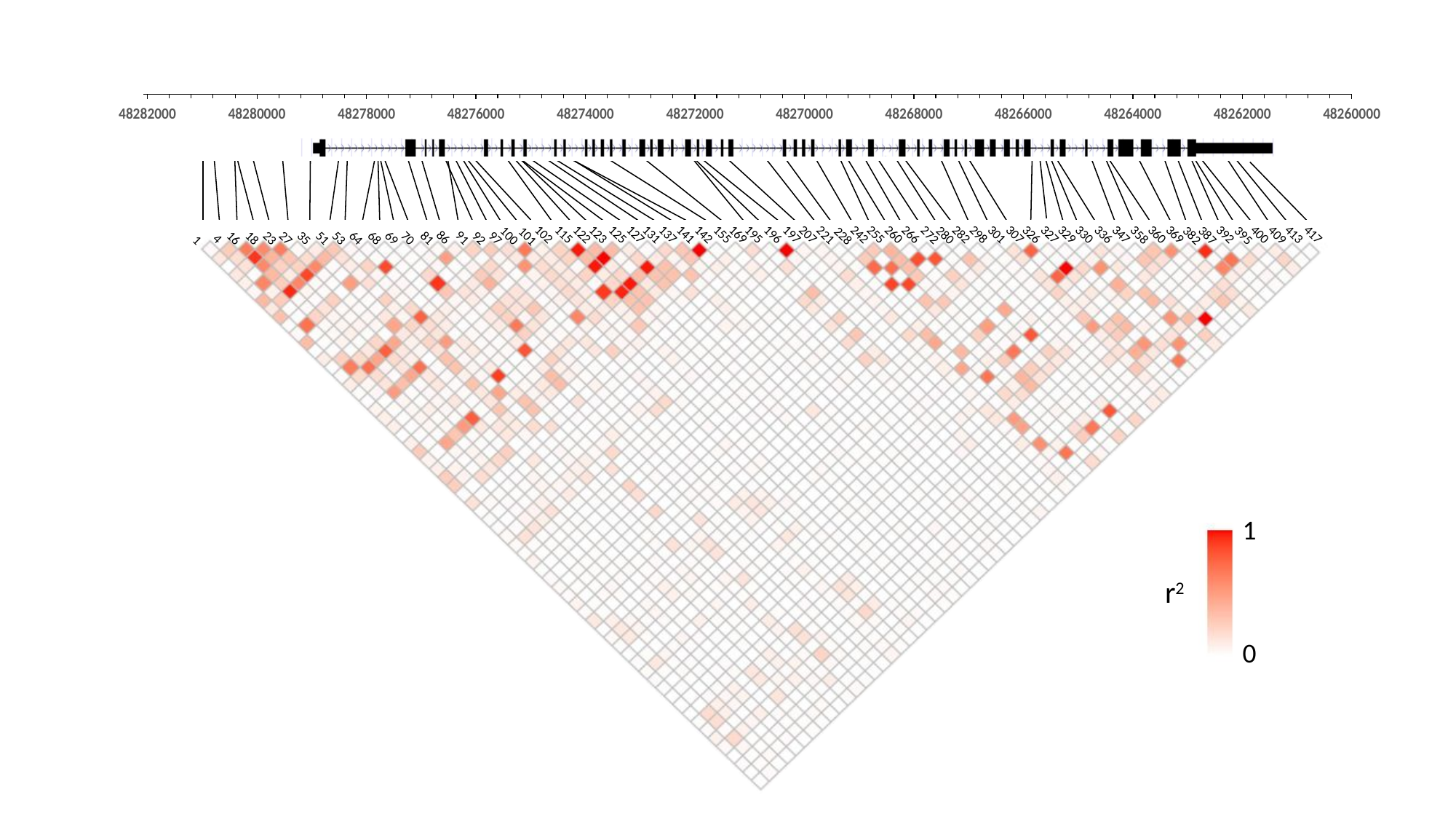

102
100
101
91
97
 86
92
115
122
123
125
127
131
137
141
142
155
169
195
196
197
207
242
255
260
266
282
298
301
307
326
327
329
330
336
347
360
369
392
400
413
417
221
409
358
272
280
387
382
395
228
16
18
23
27
35
51
53
64
68
69
70
81
4
1
1
r2
0

## Slide 11
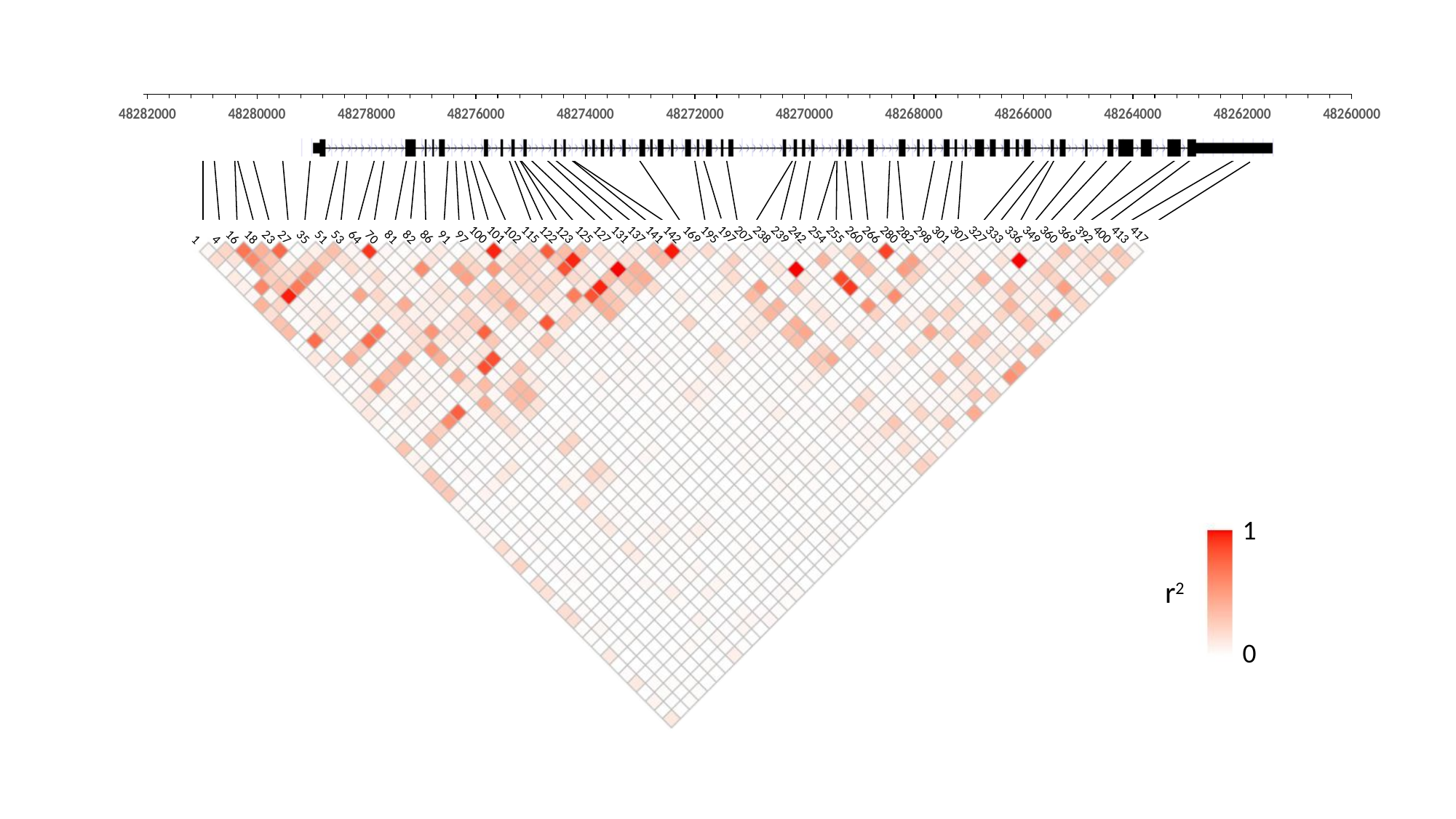

100
101
102
115
122
123
125
127
131
137
141
142
169
195
197
207
238
239
242
254
255
260
266
280
282
298
301
307
327
333
336
349
360
369
392
400
413
417
16
18
23
27
35
51
53
64
70
81
82
91
97
 86
1
4
1
r2
0

## Slide 12
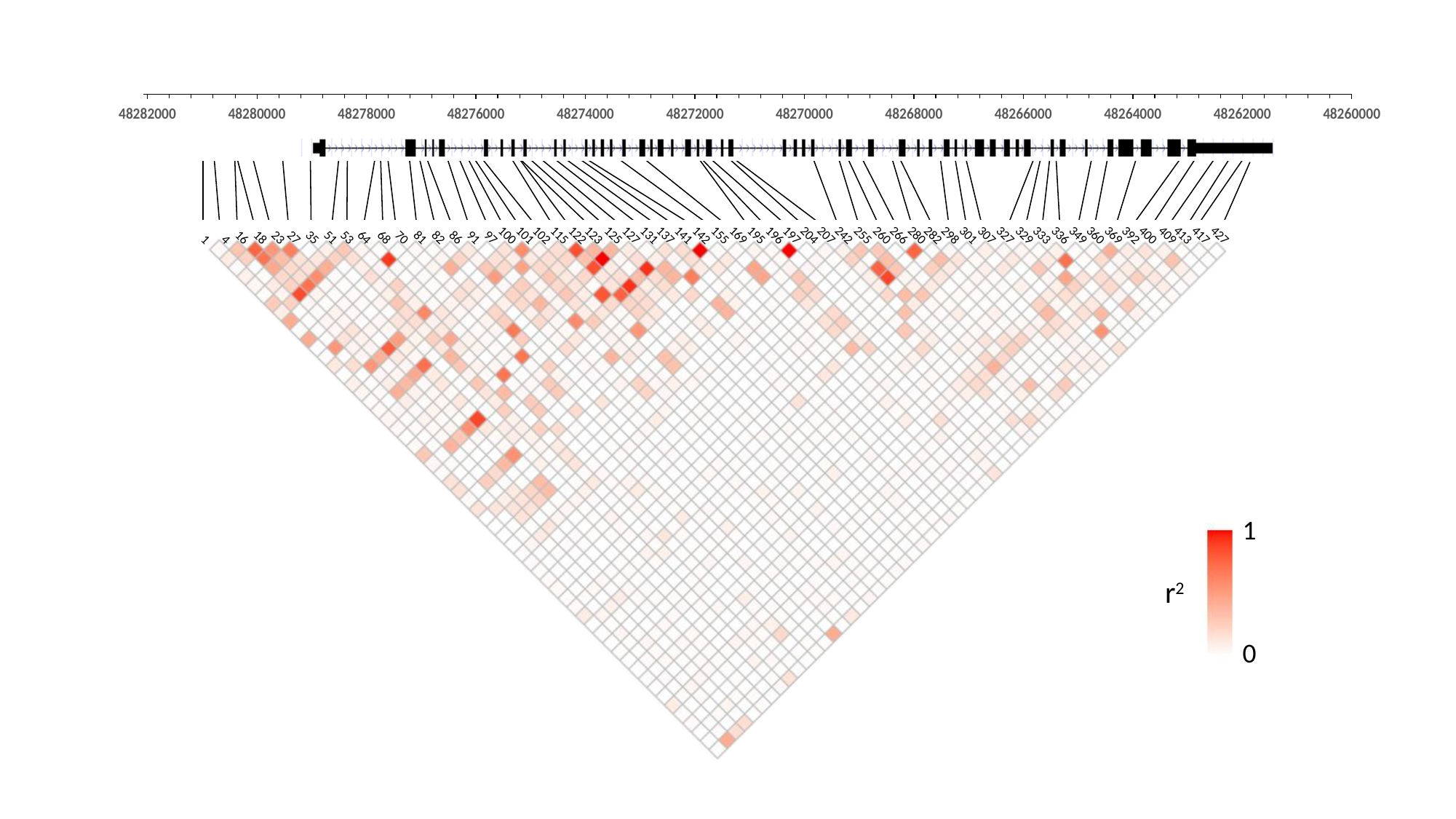

100
101
102
115
122
123
125
127
131
137
141
142
155
169
195
196
197
204
207
242
255
260
266
280
282
298
301
307
327
329
333
336
349
360
369
392
400
409
413
417
427
16
18
23
27
35
51
53
64
68
70
81
82
86
91
97
1
4
1
r2
0

## Slide 13
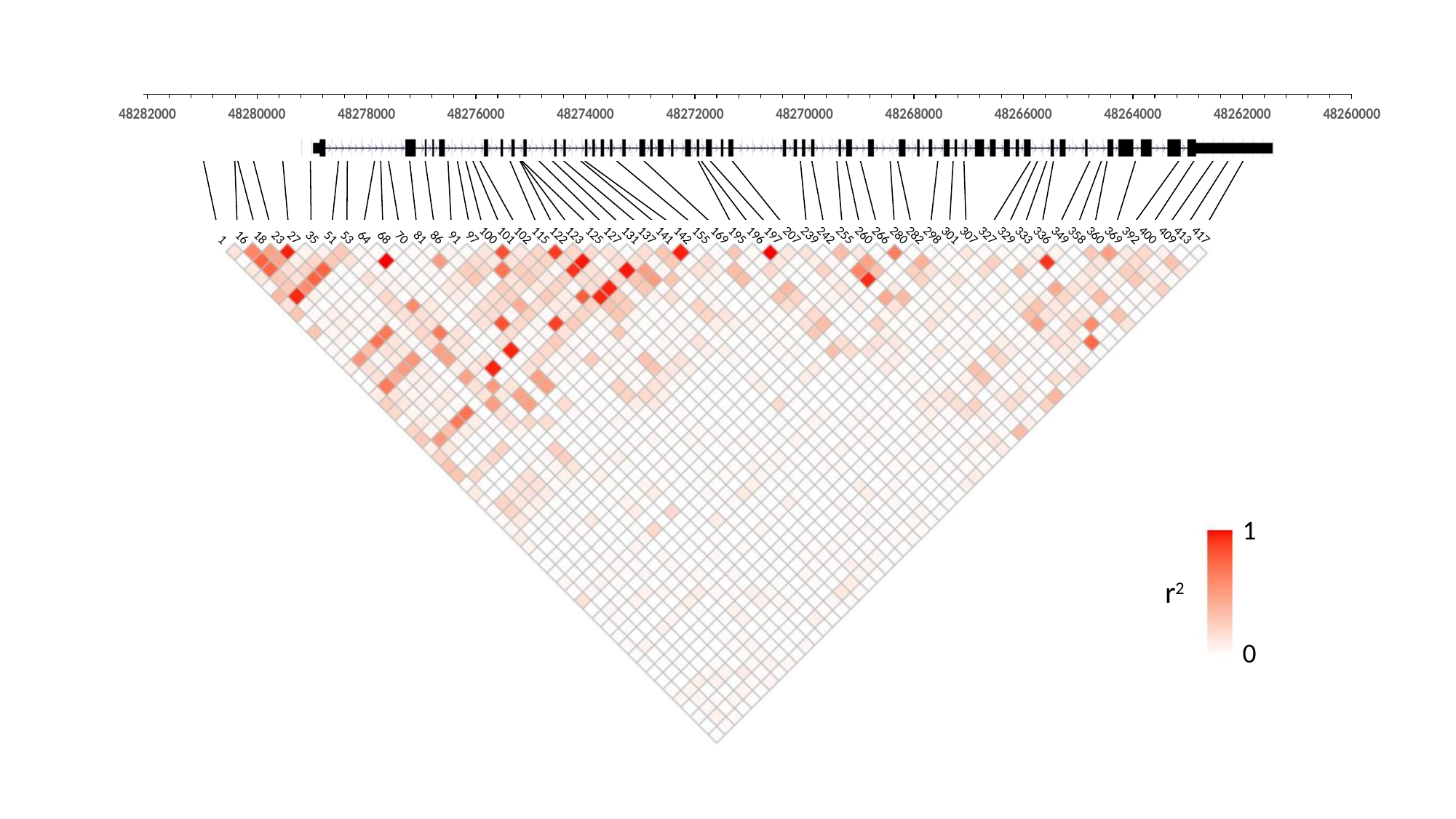

100
101
102
115
122
123
125
127
131
137
141
142
155
169
195
196
197
207
239
242
255
260
266
280
282
298
301
307
327
329
333
336
349
358
360
369
392
400
409
413
417
16
18
23
27
35
51
53
64
68
70
81
86
91
97
1
1
r2
0

## Slide 14
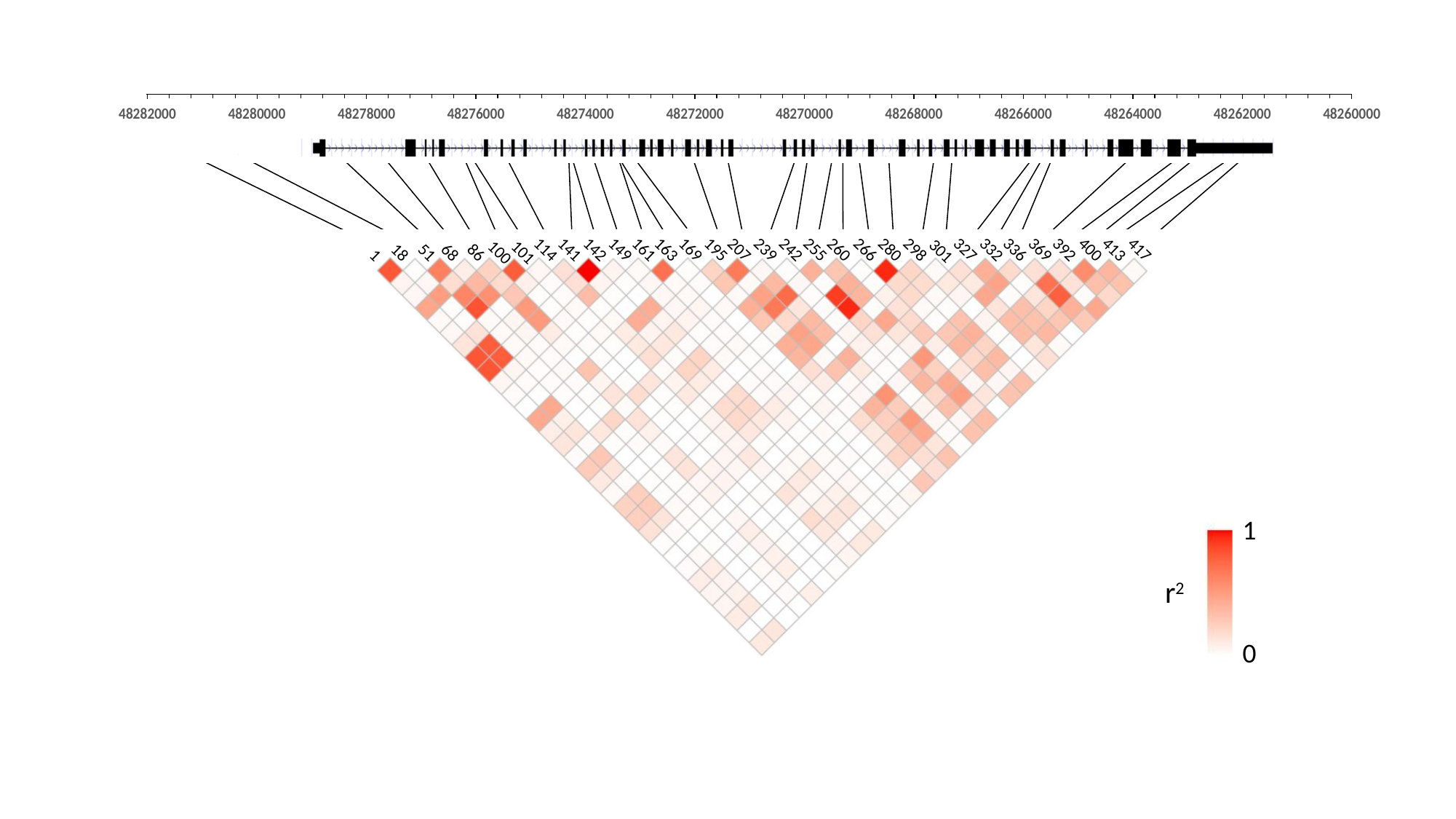

114
141
142
149
161
163
169
195
207
239
242
255
260
266
280
298
327
332
336
369
392
400
413
417
301
18
51
68
86
100
101
1
1
r2
0

## Slide 15
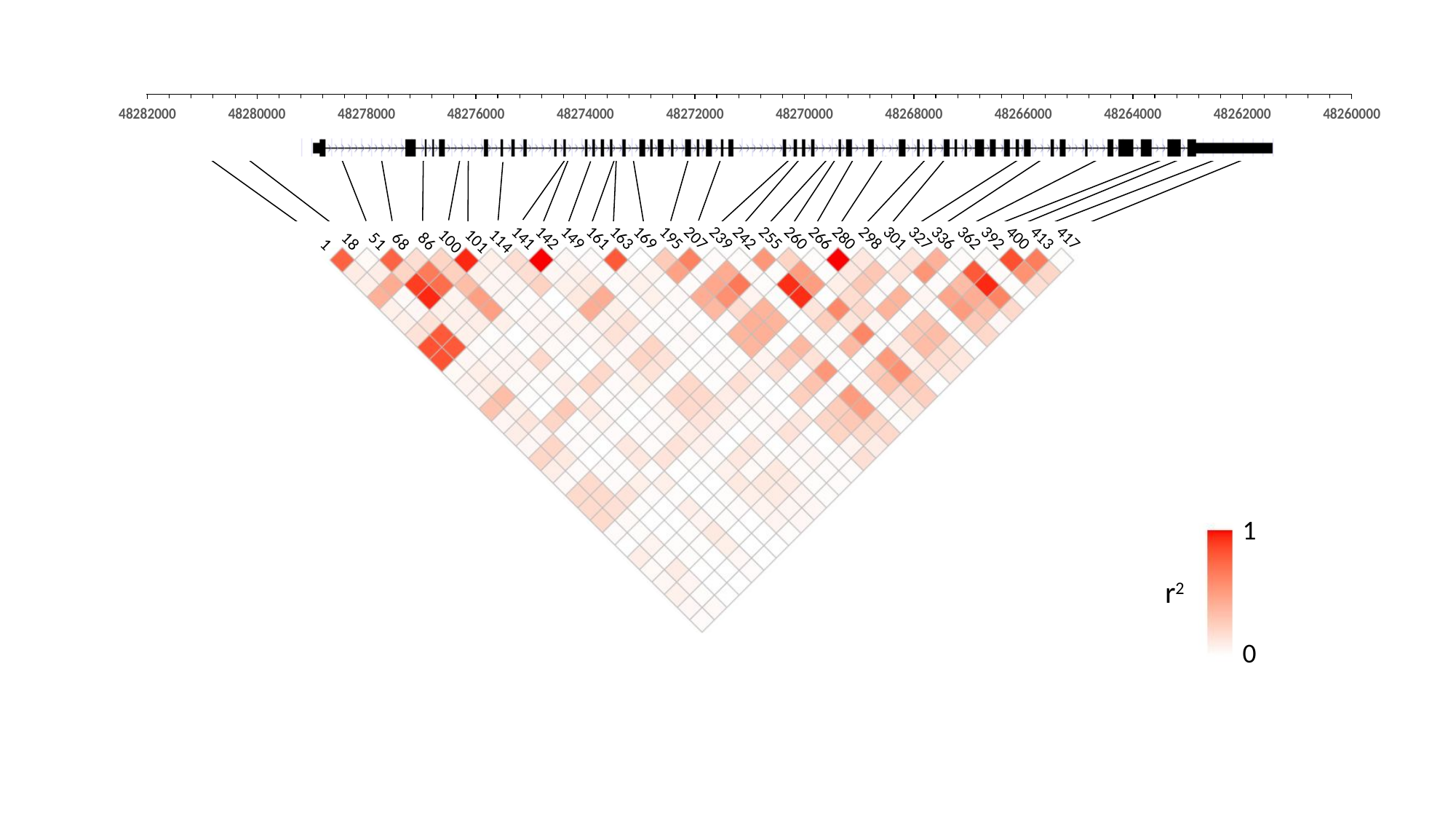

141
142
149
161
163
169
195
207
239
242
255
260
266
280
298
301
327
336
362
392
400
413
417
18
51
68
86
100
101
114
1
1
r2
0

## Slide 16
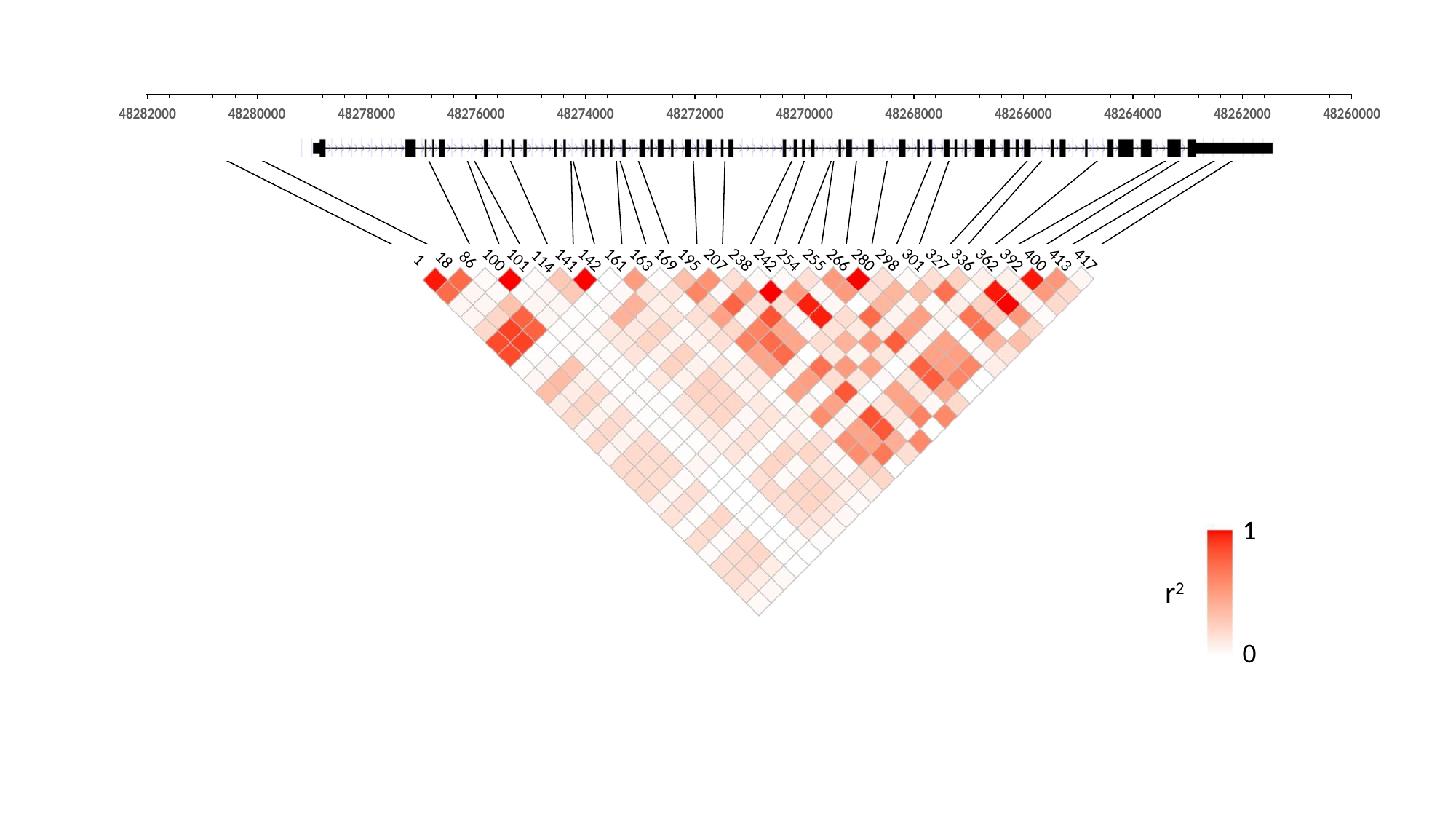

1
18
86
100
101
141
142
161
163
169
195
207
238
242
254
255
266
280
298
301
327
336
362
392
400
413
417
114
1
r2
0

## Slide 17
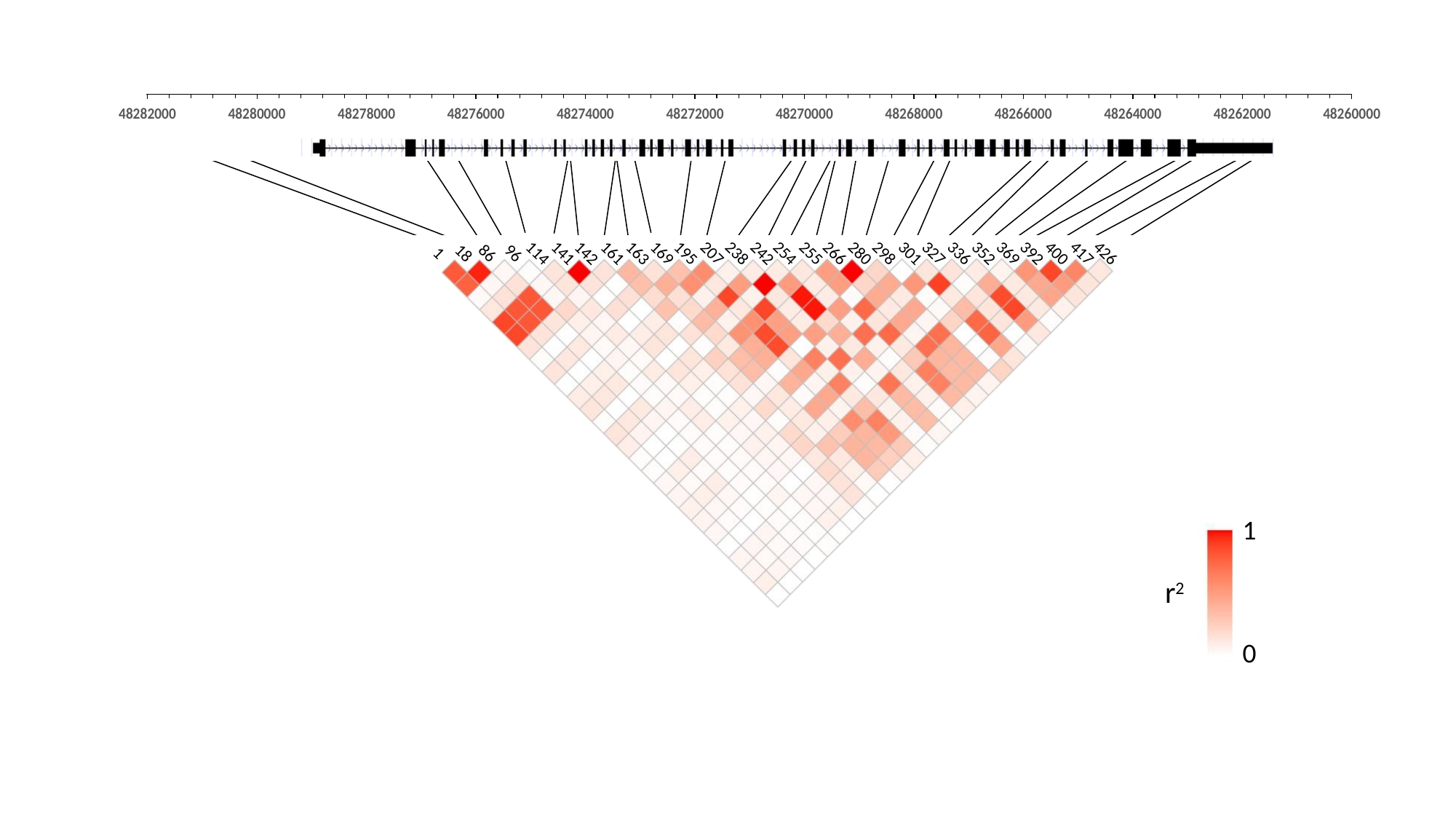

1
18
86
96
114
141
142
161
163
169
195
207
238
242
254
255
266
280
298
301
327
336
352
369
392
400
417
426
1
r2
0

## Slide 18
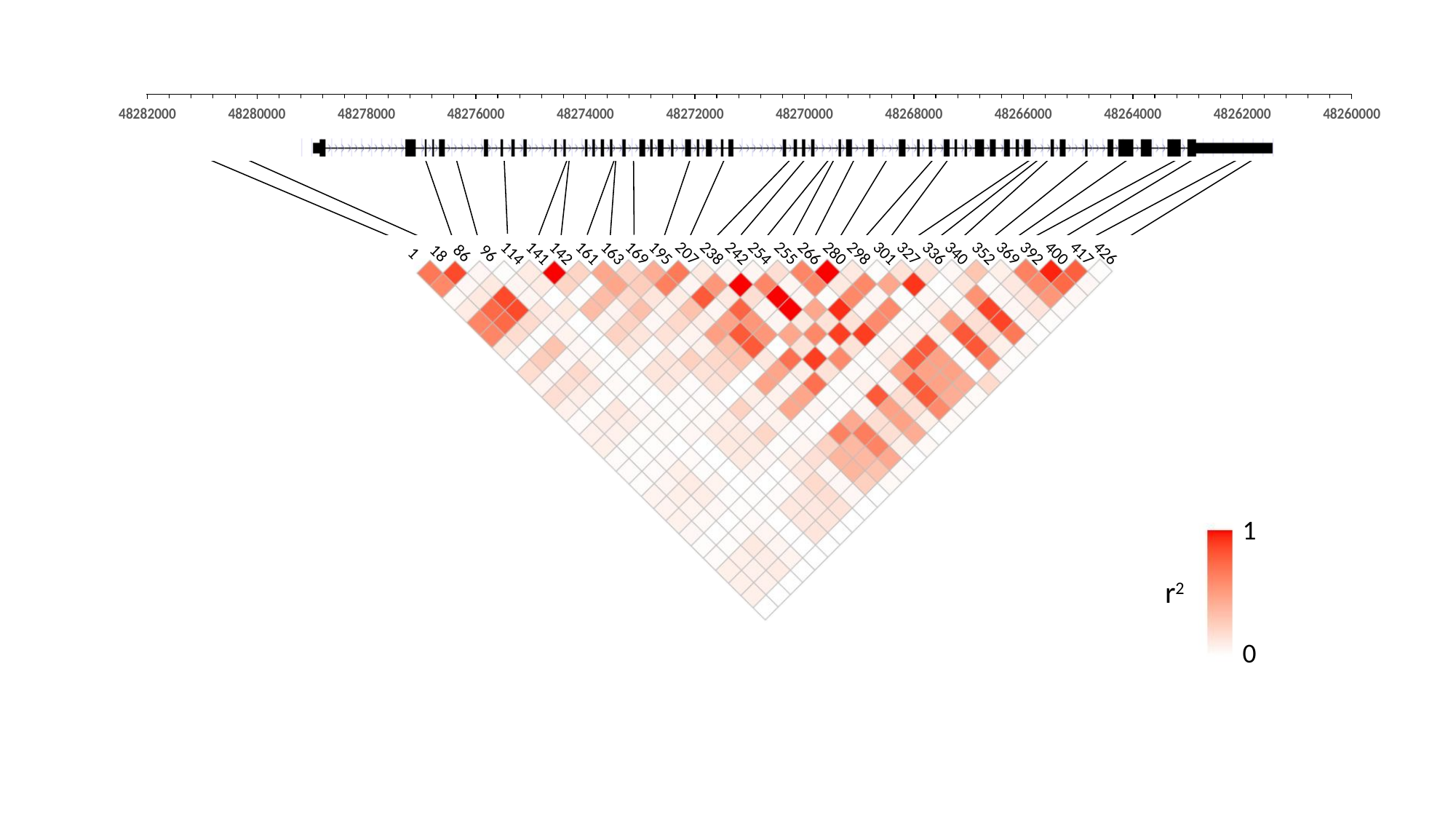

1
18
86
96
114
141
142
161
163
169
195
207
238
242
254
255
266
280
298
301
327
336
340
352
369
392
400
417
426
1
r2
0

## Slide 19
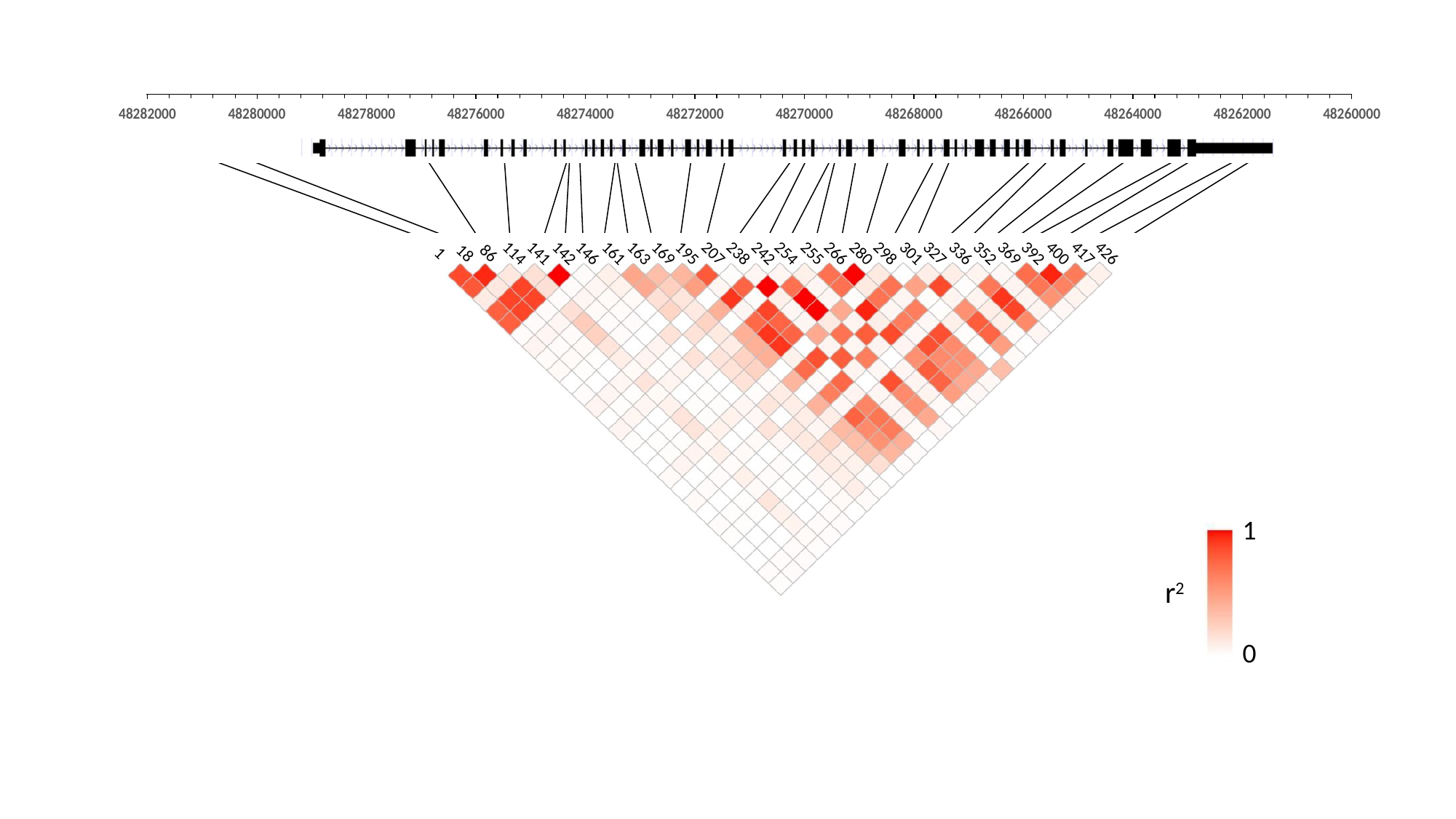

1
18
86
114
141
142
146
161
163
169
195
207
238
242
254
255
266
280
298
301
327
336
352
369
392
400
417
426
1
r2
0

## Slide 20
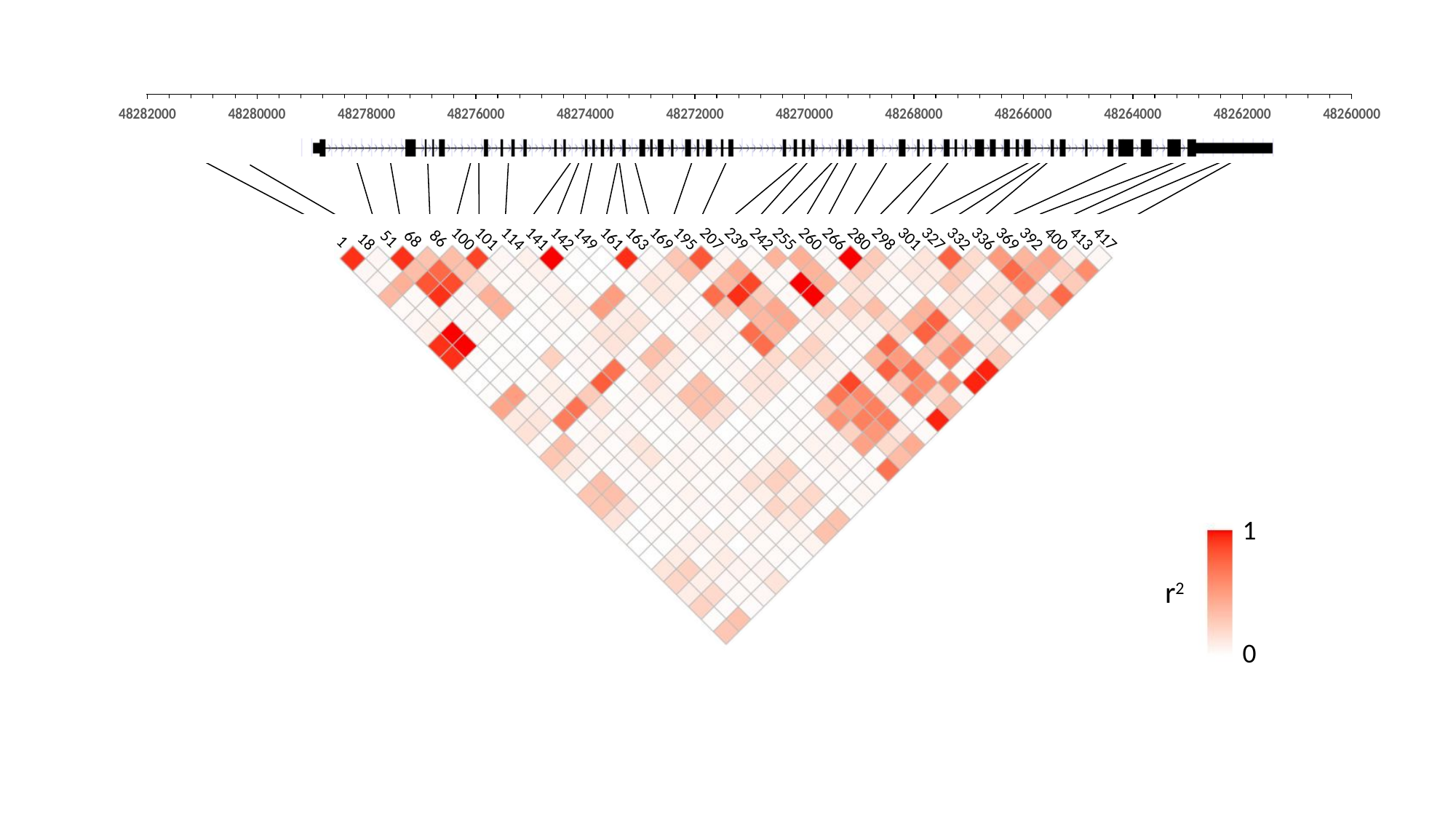

51
68
86
100
101
114
141
142
149
161
163
169
195
207
239
242
255
260
266
280
298
301
327
332
336
369
392
400
413
417
1
18
1
r2
0

## Slide 21
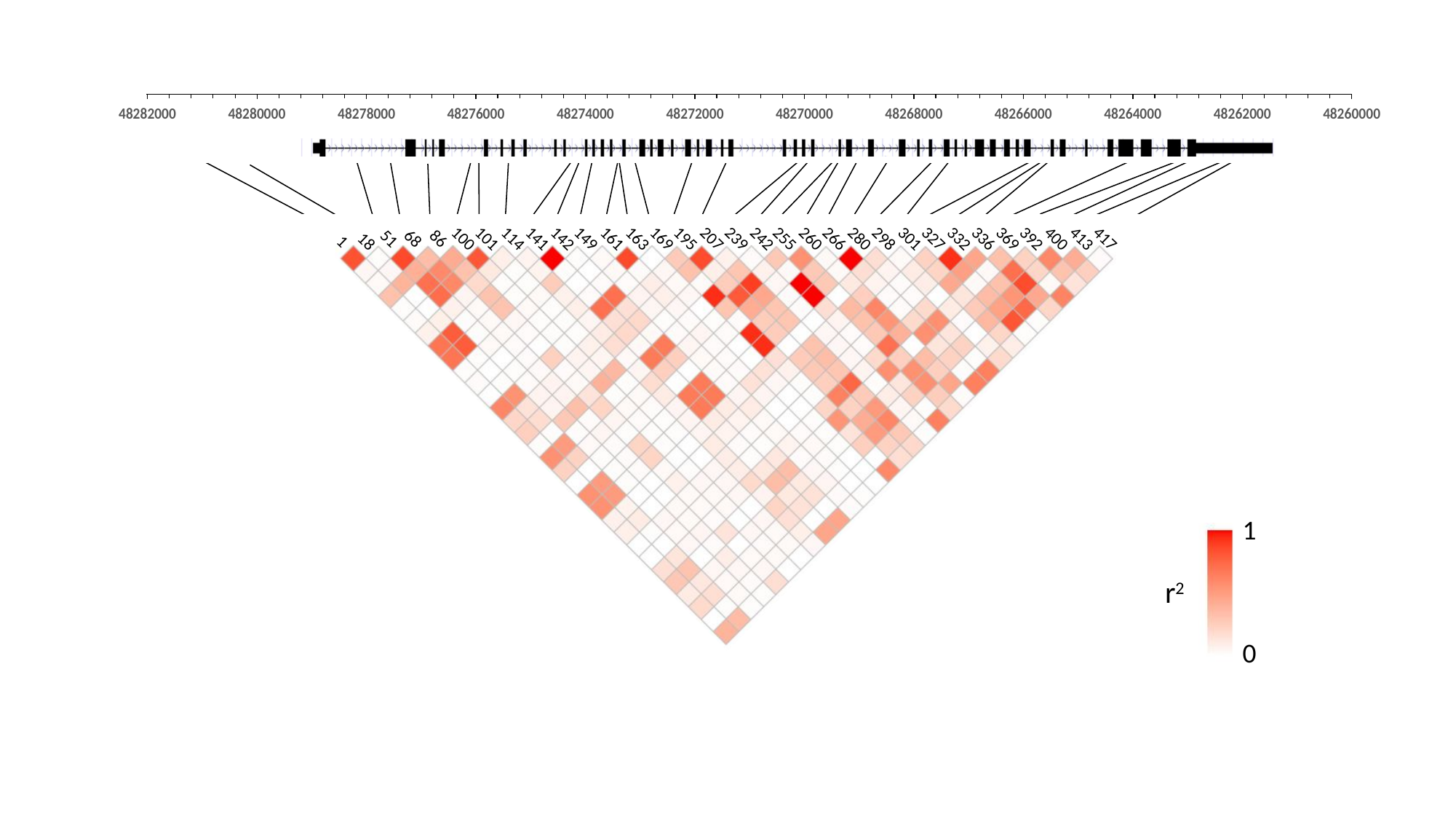

51
68
86
100
101
114
141
142
149
161
163
169
195
207
239
242
255
260
266
280
298
301
327
332
336
369
392
400
413
417
1
18
1
r2
0

## Slide 22
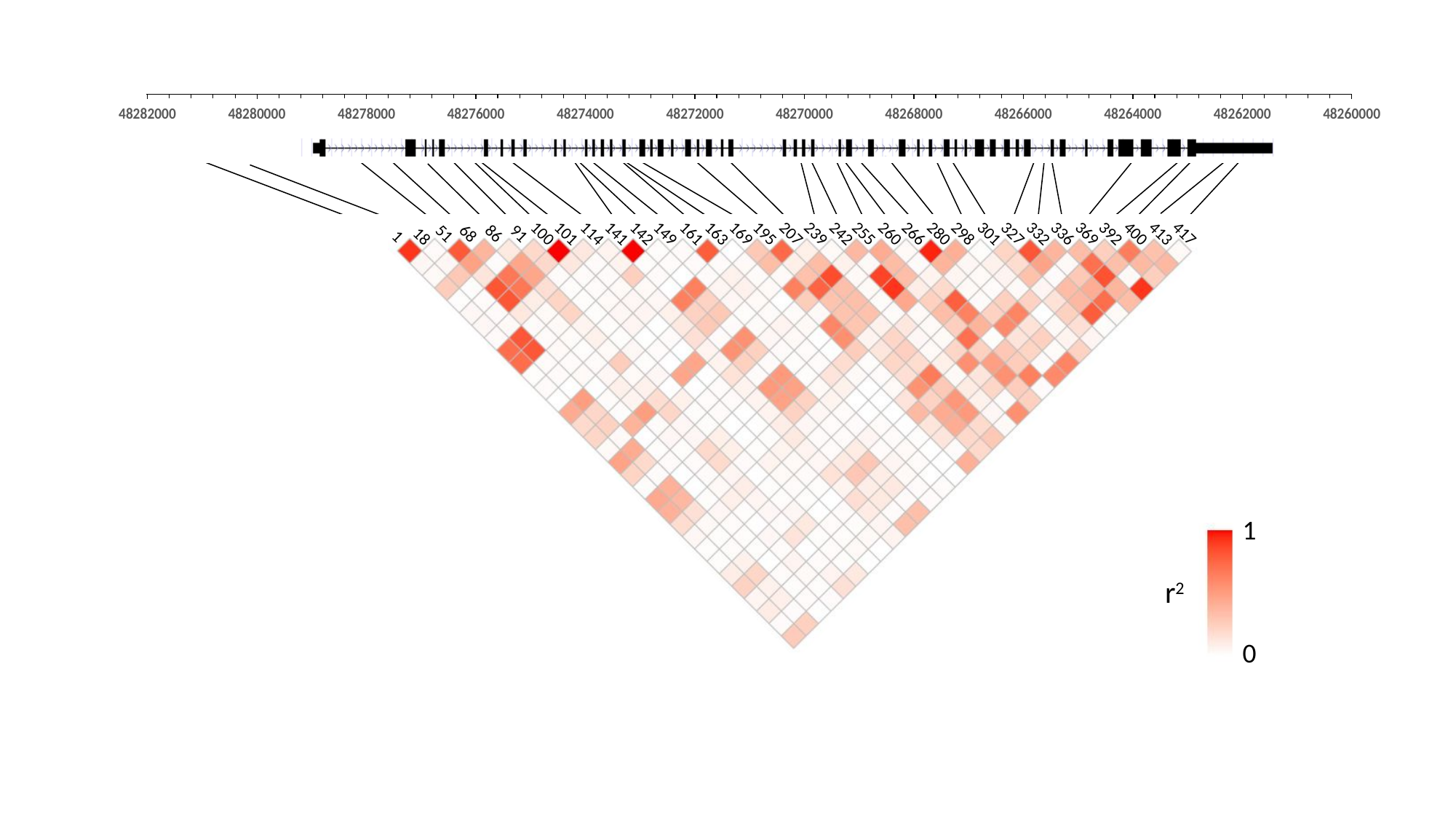

51
68
86
91
100
101
114
141
142
149
161
163
169
195
207
239
242
255
260
266
280
298
301
327
332
336
369
392
400
413
417
1
18
1
r2
0

## Slide 23
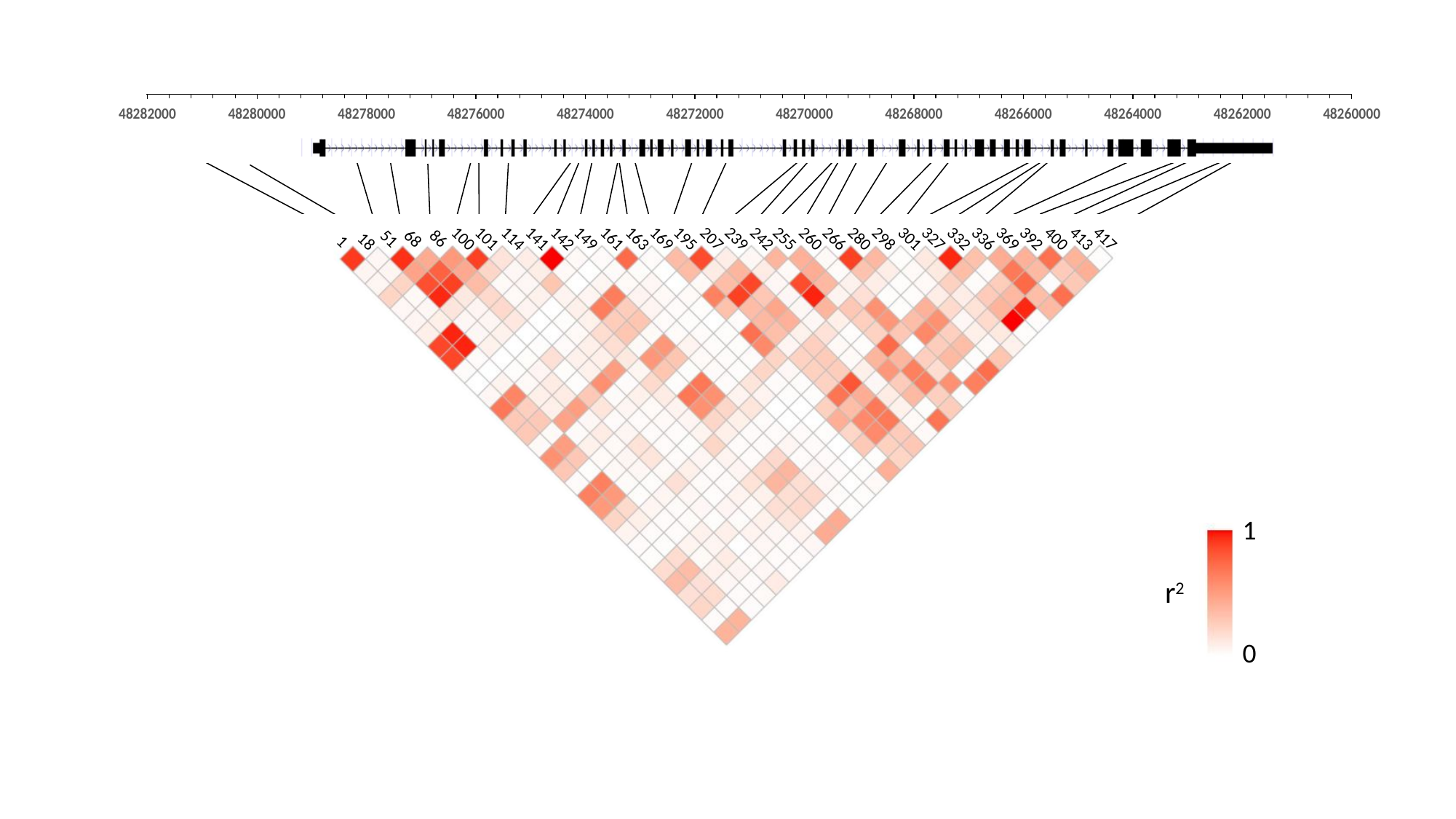

51
68
86
100
101
114
141
142
149
161
163
169
195
207
239
242
255
260
266
280
298
301
327
332
336
369
392
400
413
417
1
18
1
r2
0

## Slide 24
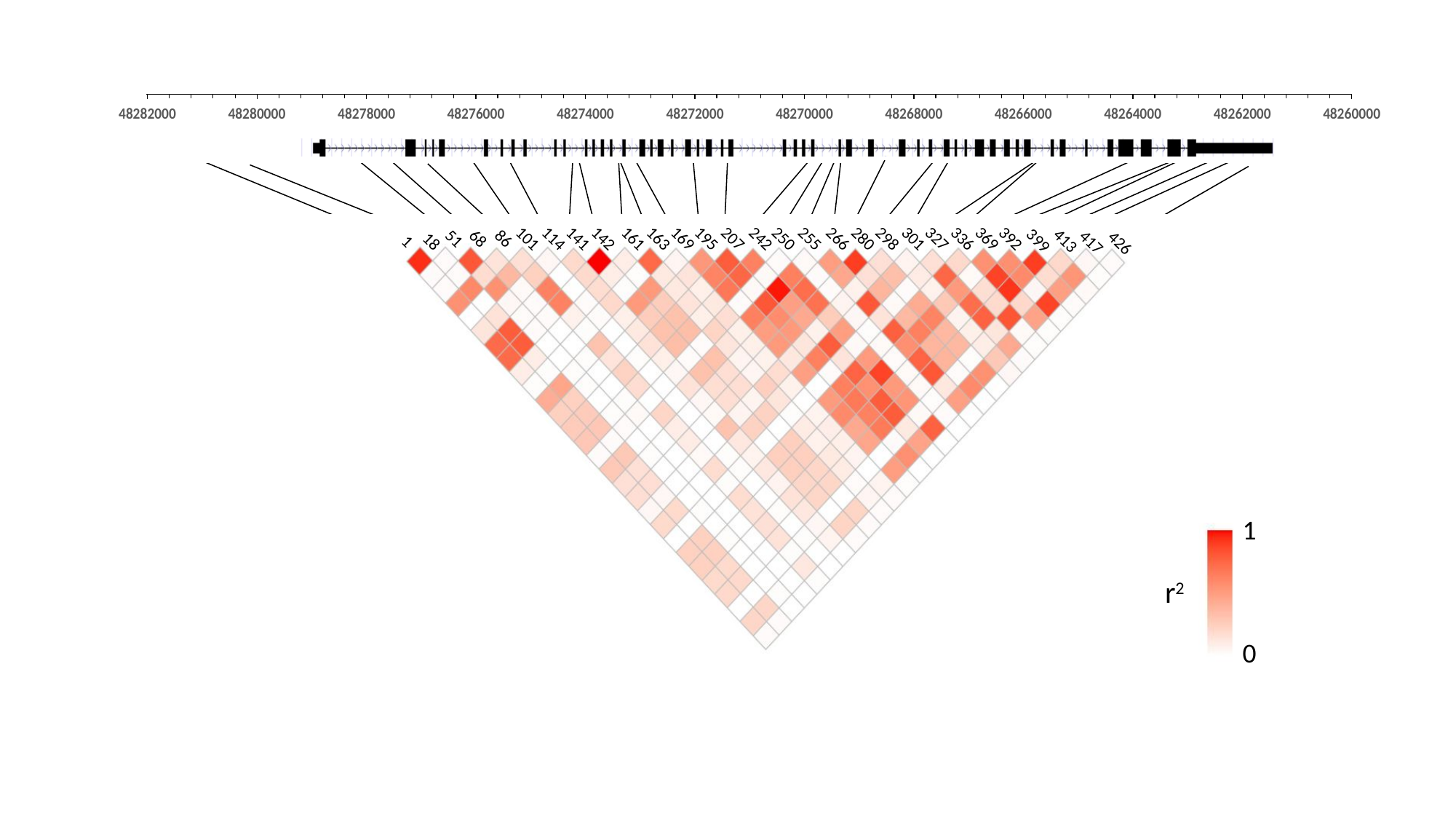

51
68
86
101
114
141
142
161
163
169
195
207
242
250
255
266
280
298
301
327
336
369
392
399
413
1
18
417
426
1
r2
0

## Slide 25
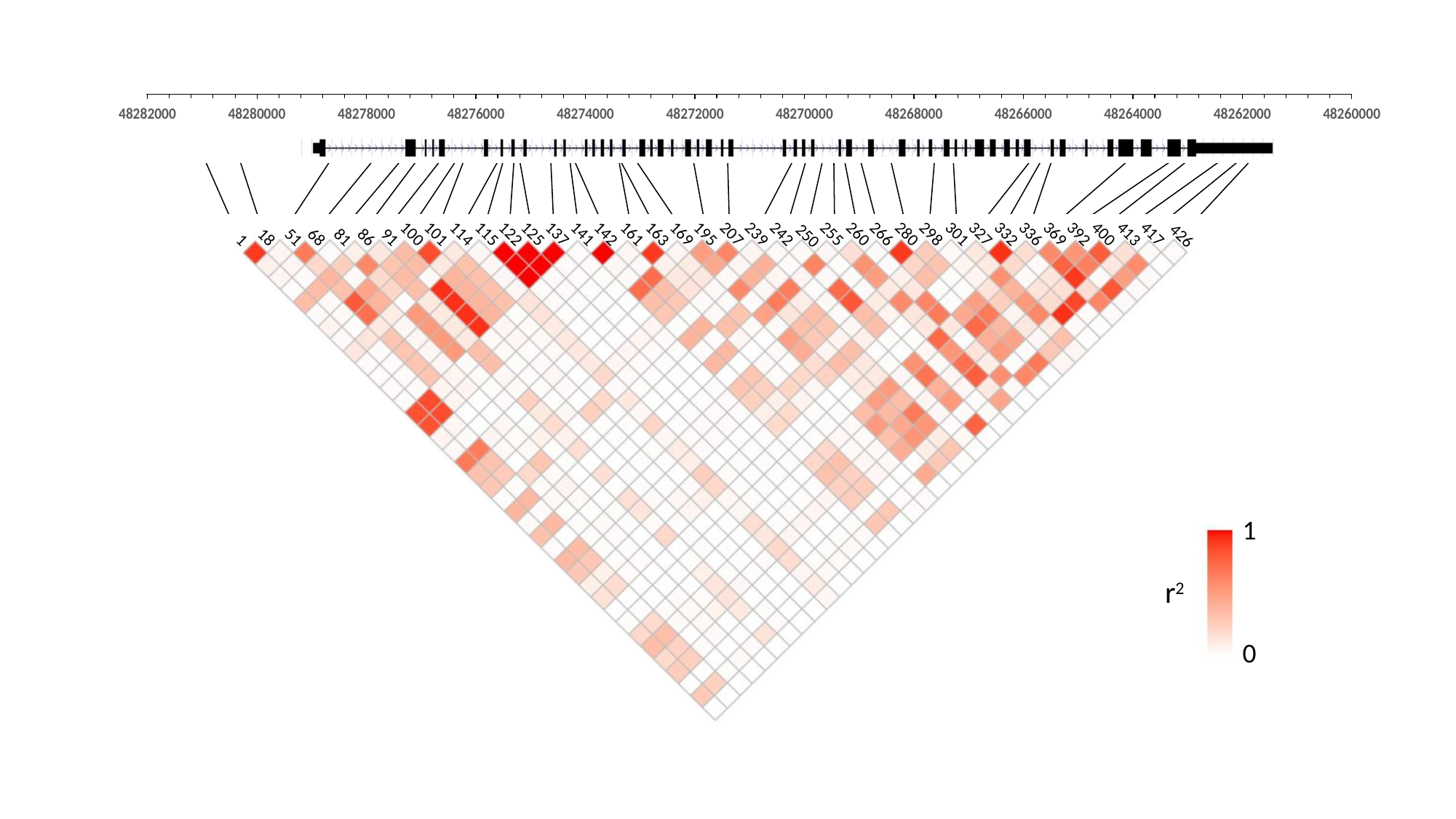

100
101
114
115
122
125
137
141
142
161
163
169
195
207
239
242
255
260
266
280
298
301
327
332
336
369
392
400
413
417
250
426
18
51
68
81
86
91
1
1
r2
0

## Slide 26
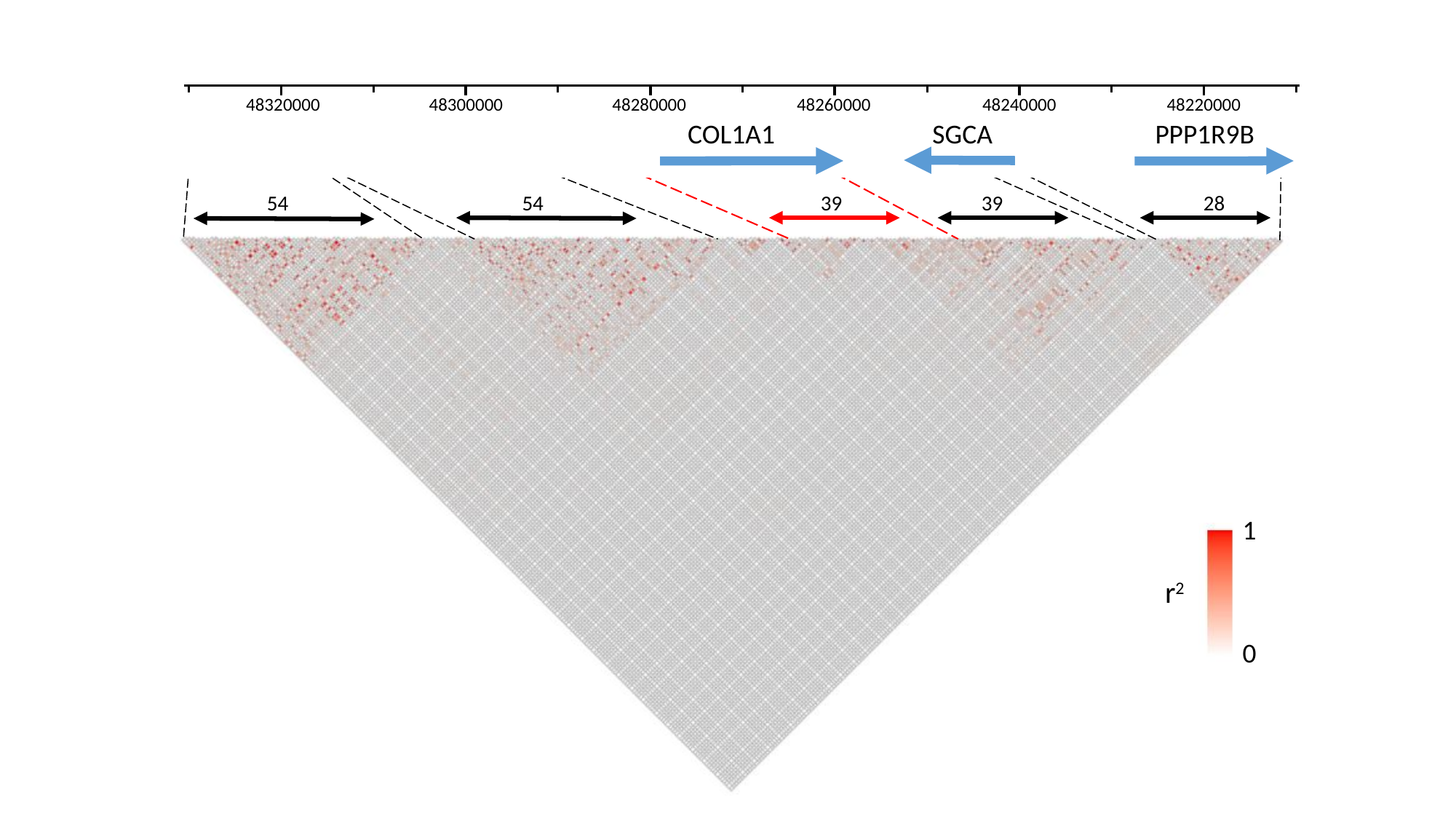

48320000
48300000
48280000
48260000
48240000
48220000
COL1A1
SGCA
PPP1R9B
54
54
39
39
28
1
r2
0

## Slide 27
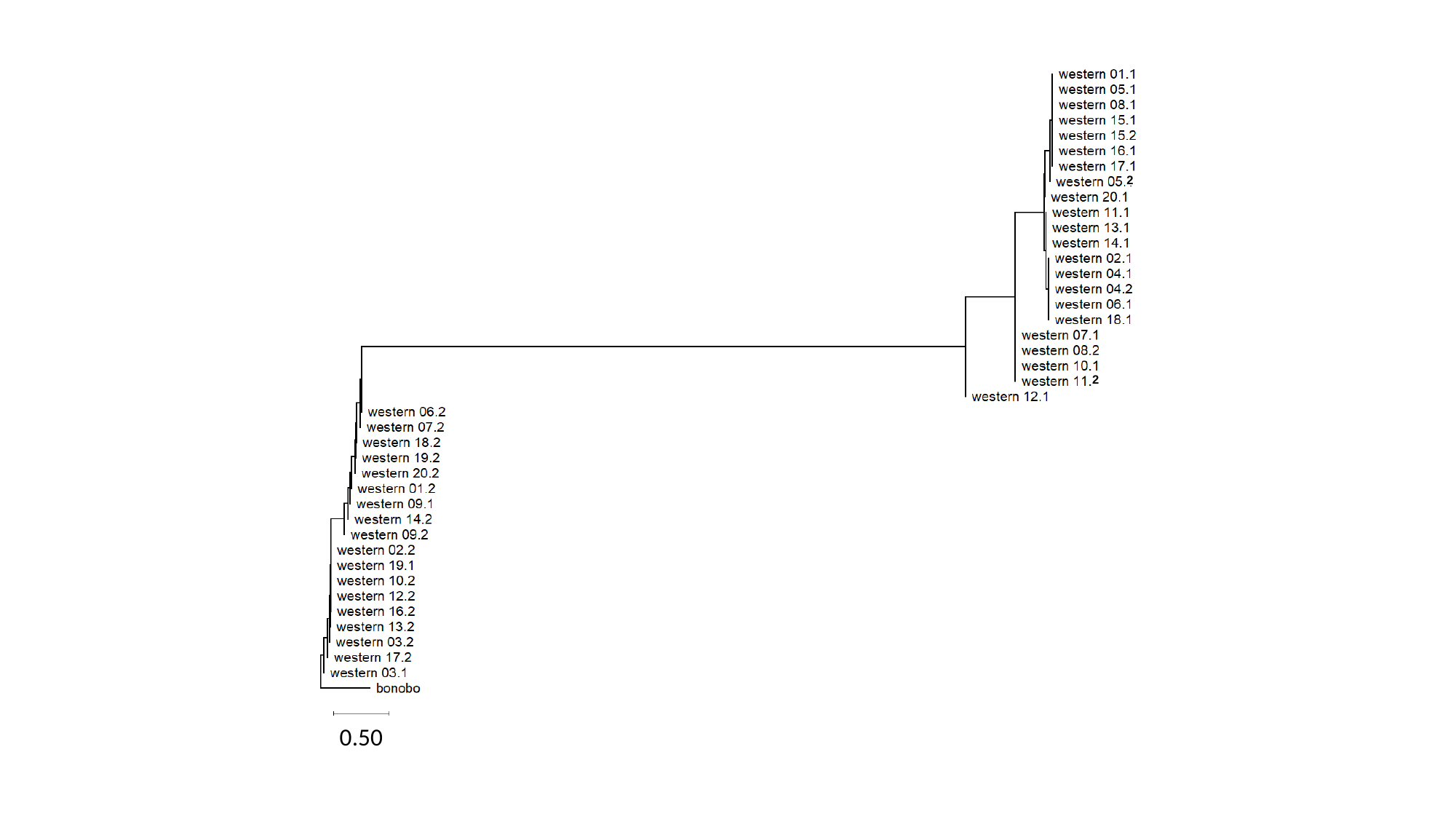

## Slide 28
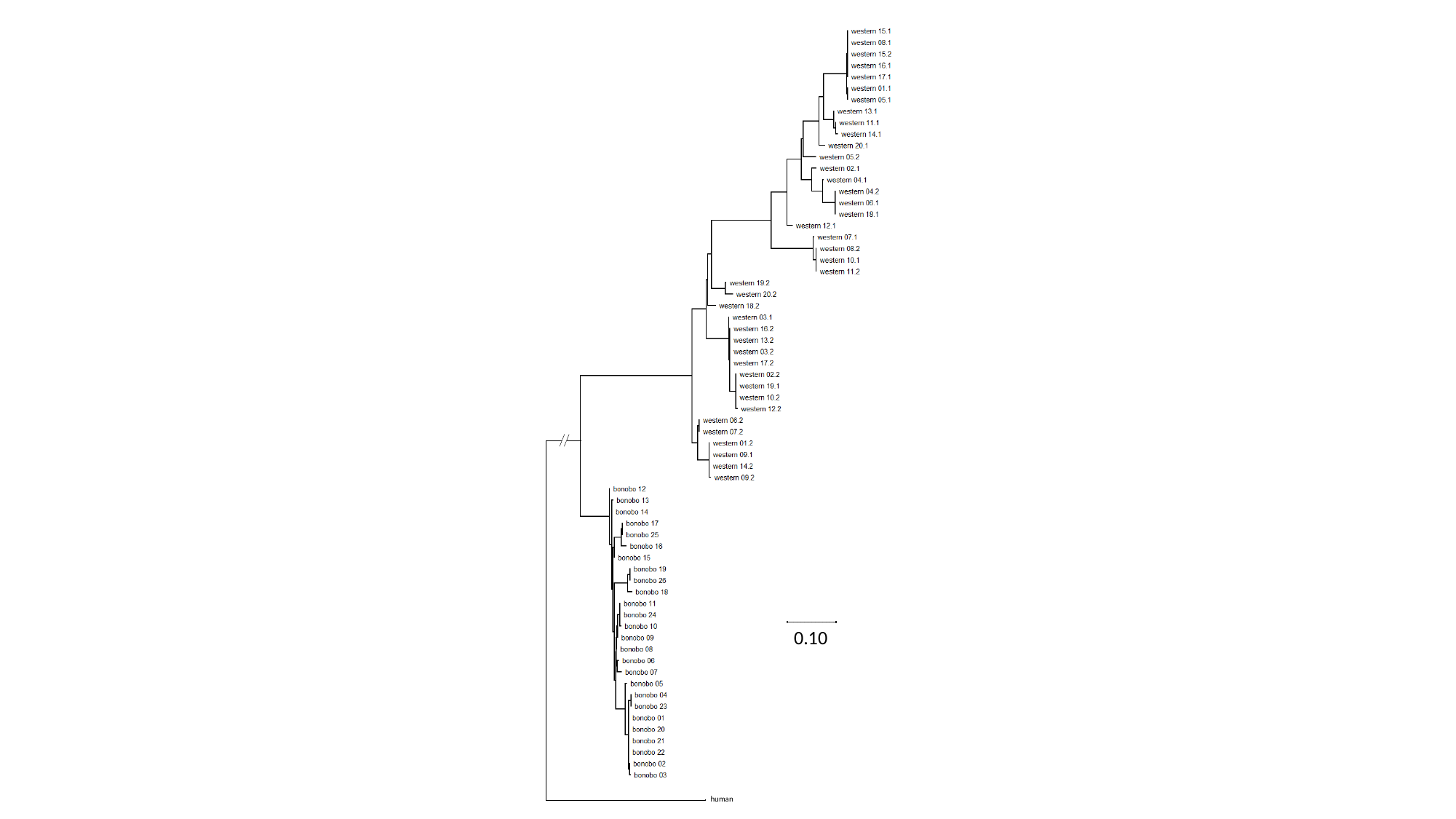

## Slide 29
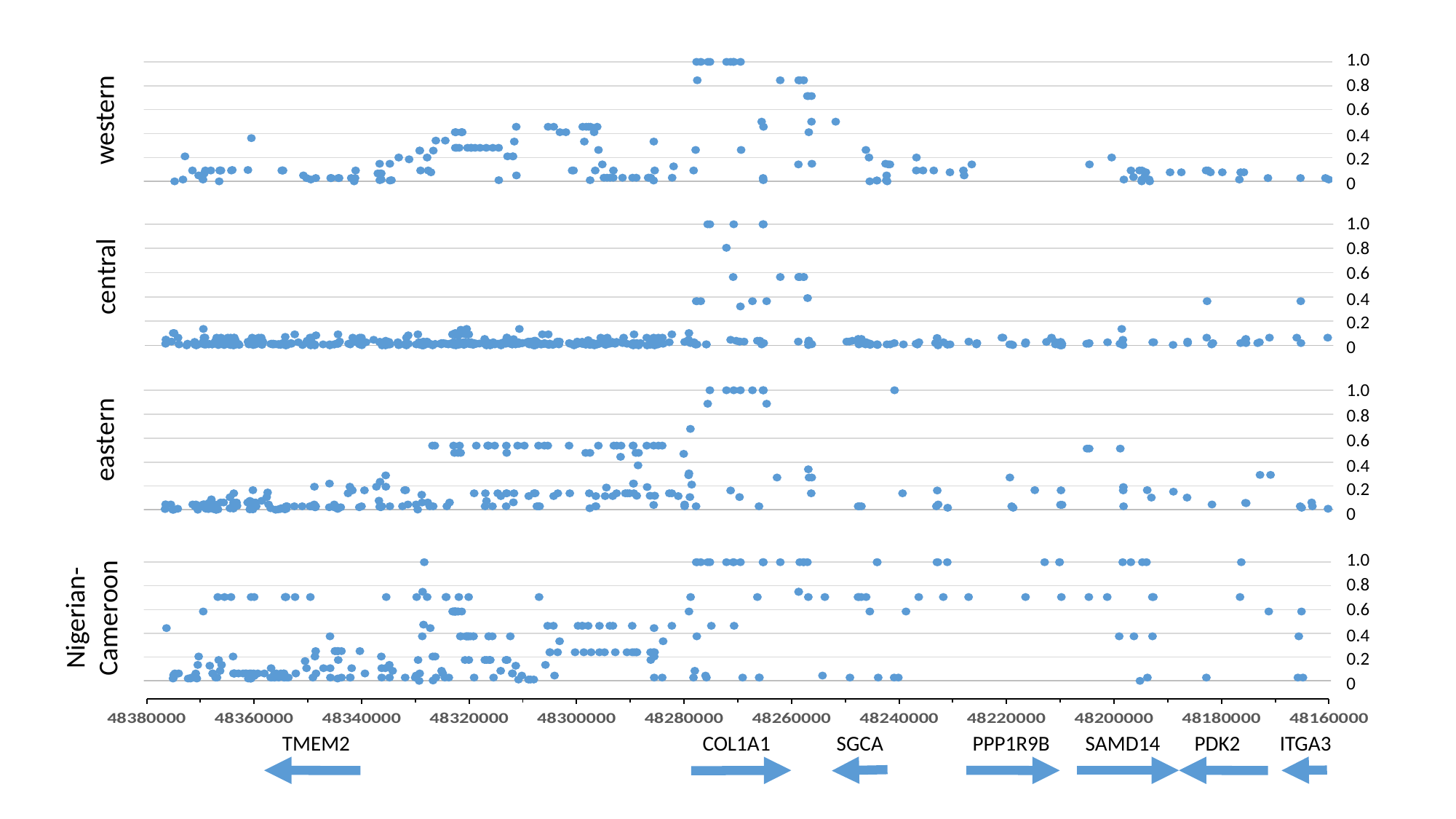

1.0
0.8
0.6
0.4
0.2
0
western
1.0
0.8
0.6
0.4
0.2
0
central
1.0
0.8
0.6
0.4
0.2
0
eastern
1.0
0.8
0.6
0.4
0.2
0
Nigerian-
Cameroon
TMEM2
COL1A1
SGCA
PPP1R9B
SAMD14
PDK2
ITGA3

## Slide 30
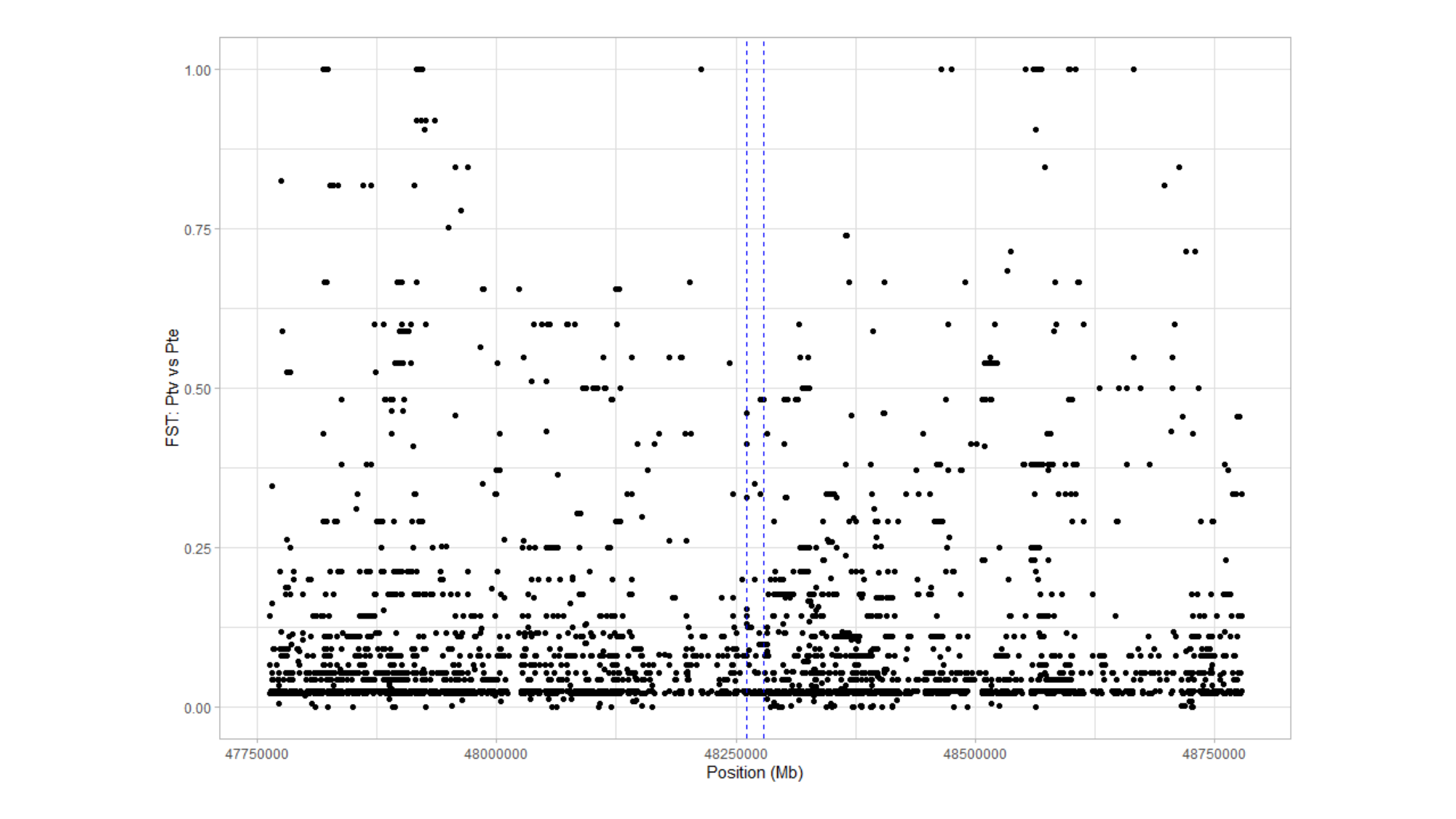

## Slide 31
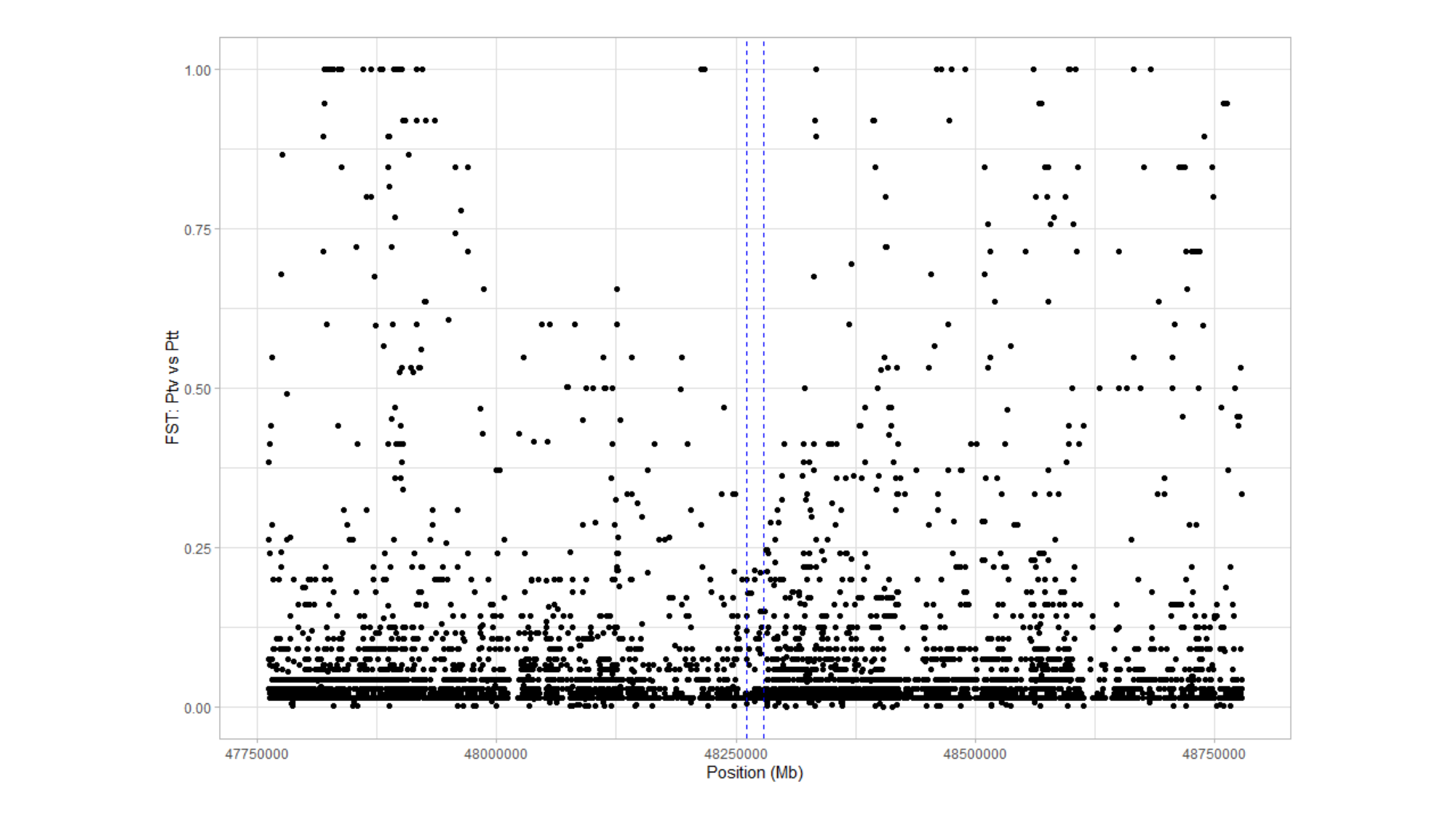

## Slide 32
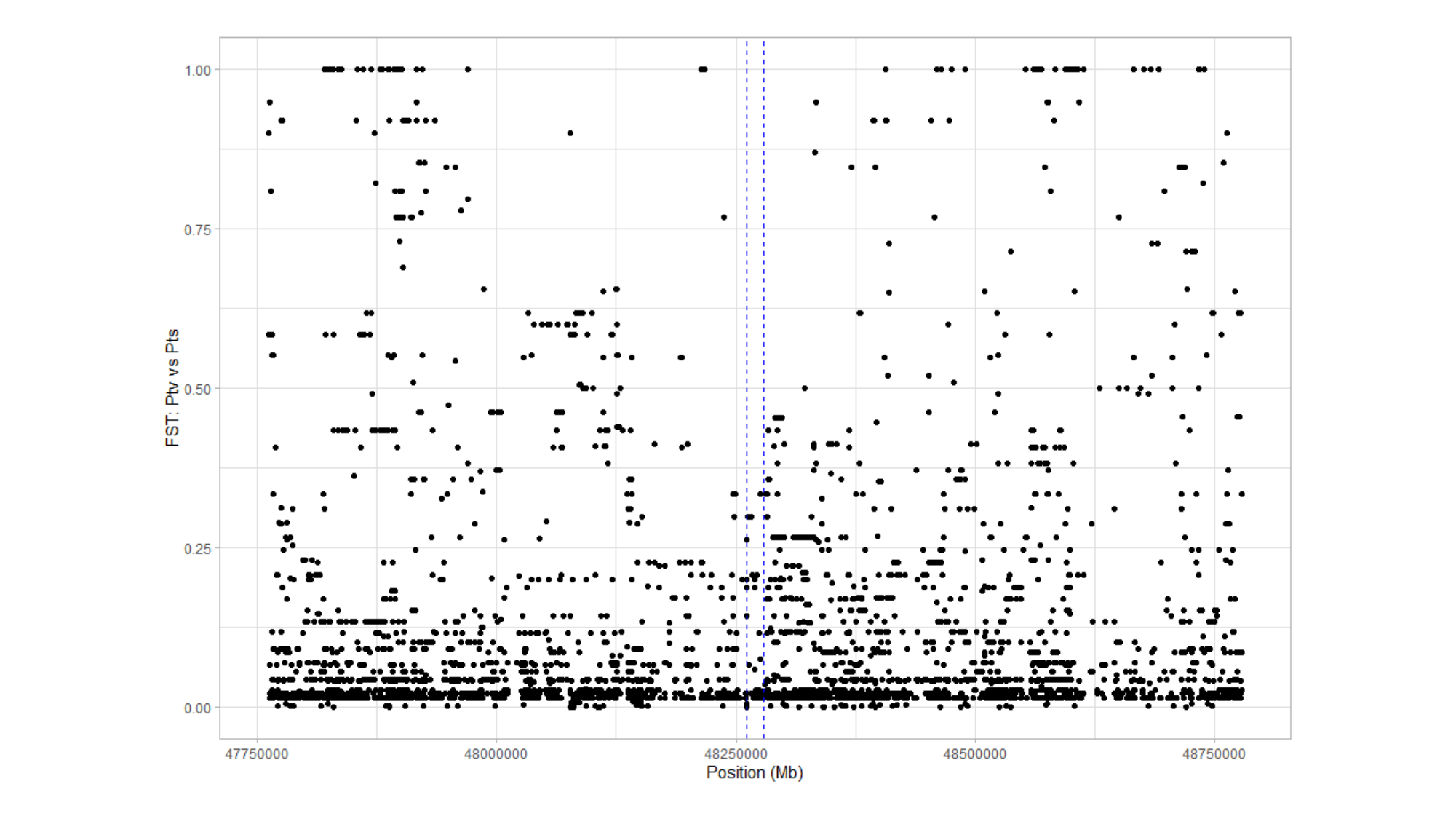

## Slide 33
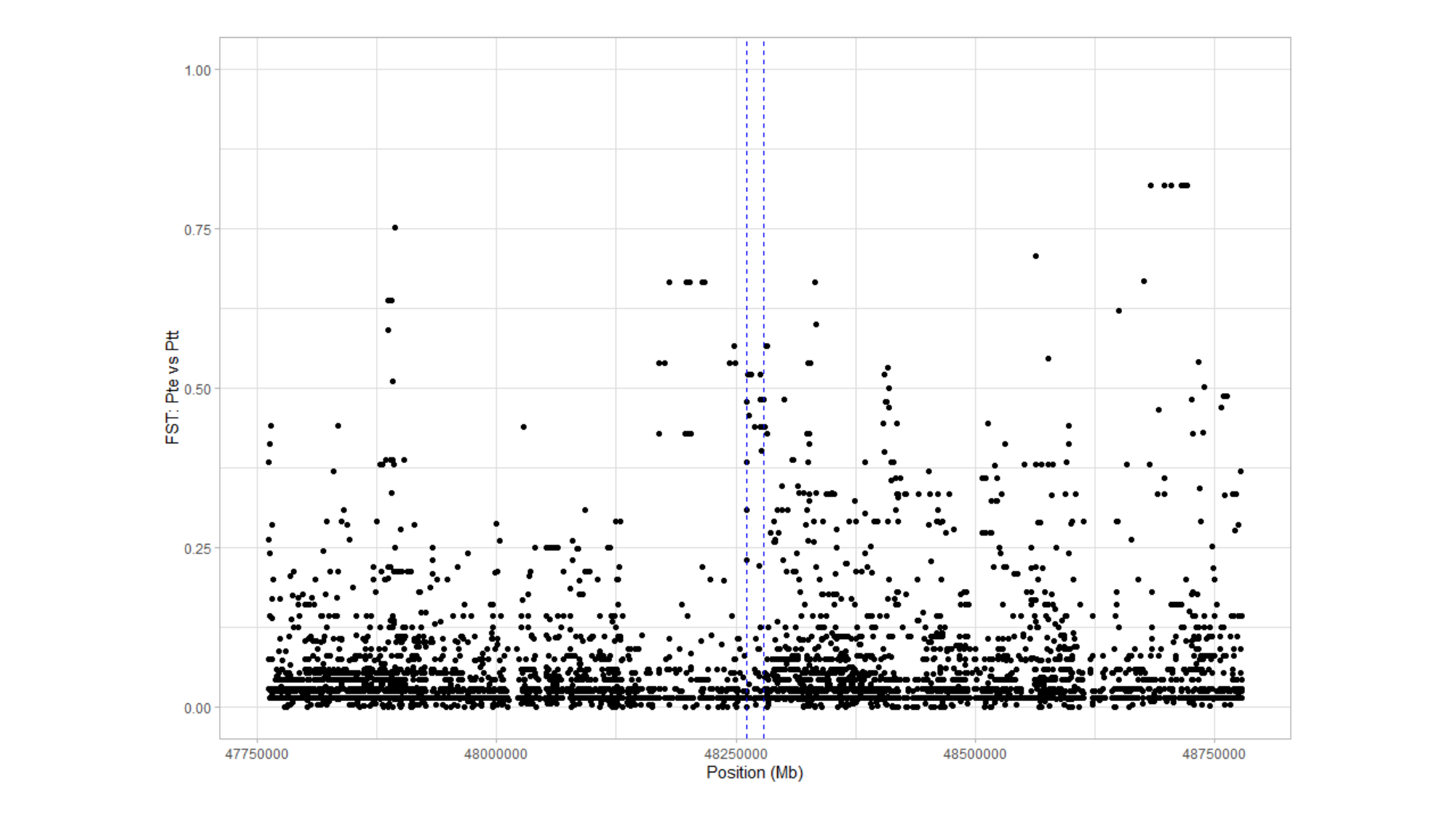

## Slide 34
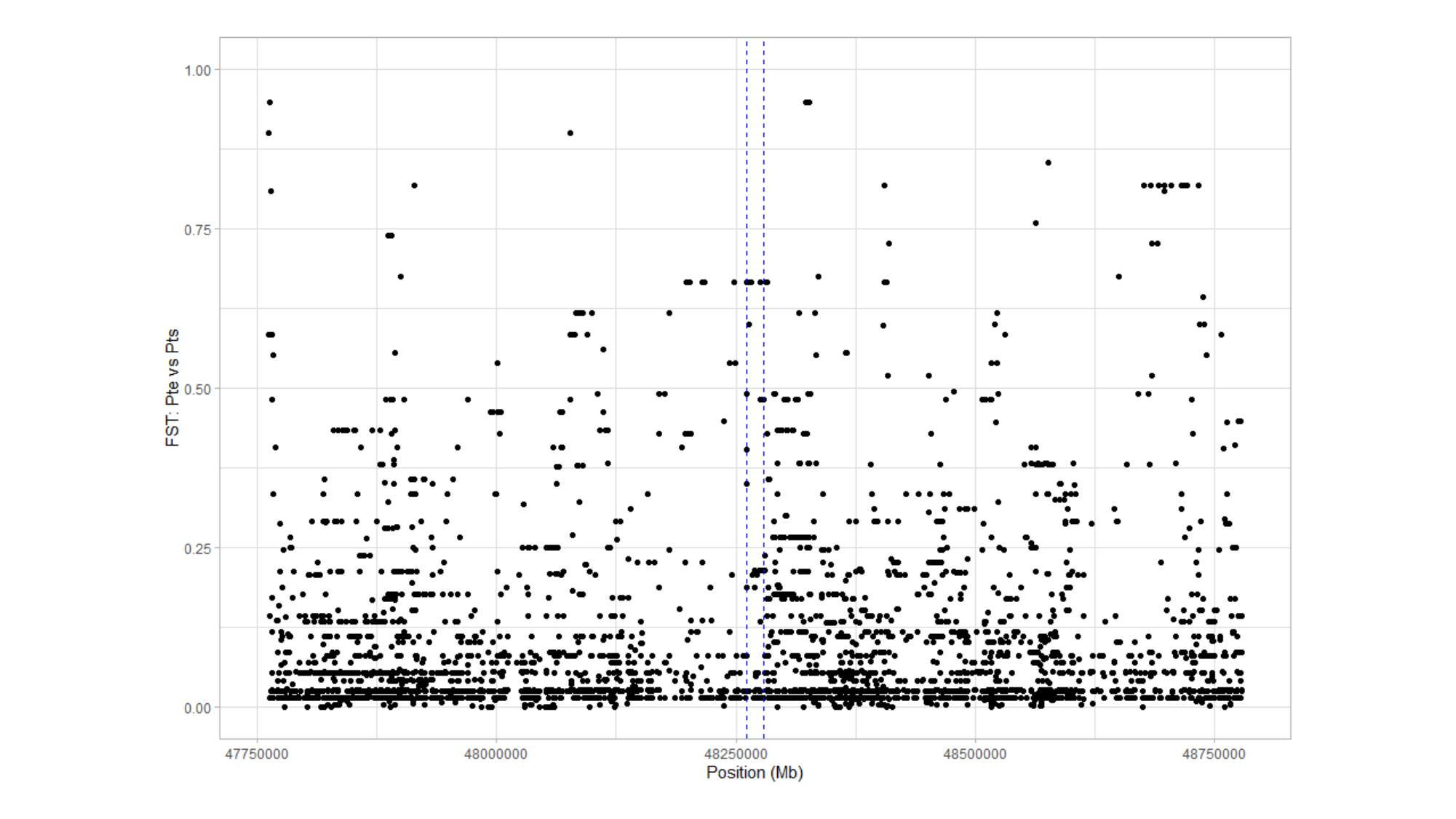

## Slide 35
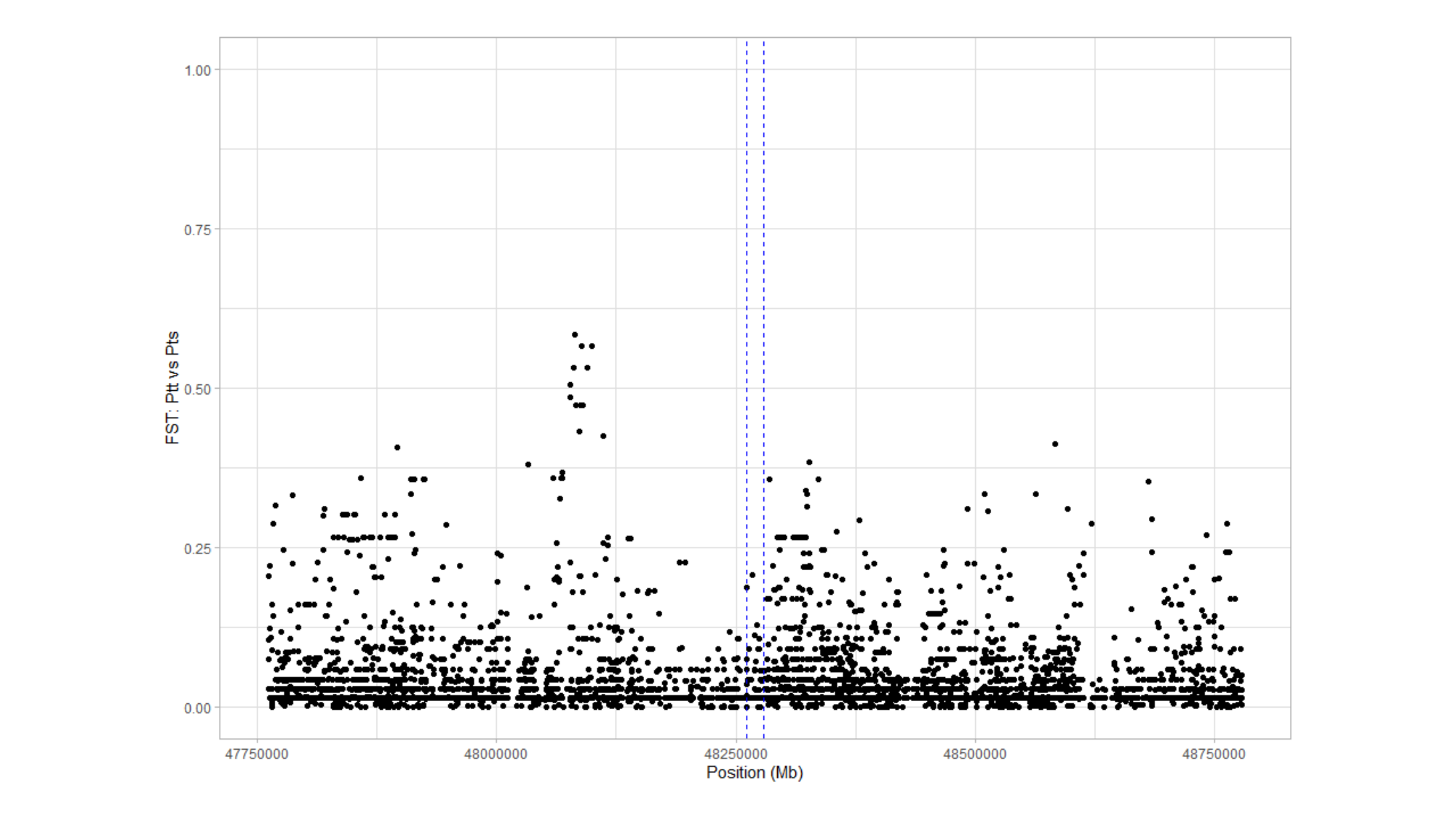

Supplement: Supplementary file 1 [file genes-13-00183-s001.zip › Stover et al_GENES_COL1A1_Supp Figures S1-S12_final.pptx]
