# Supplementary material for: Evolutionary Genetic Signatures of Selection on Bone-Related Variation within Human and Chimpanzee Populations
Source: Genes (Basel). 2022 Jan 21;13(2):183. doi: 10.3390/genes13020183 (PMC8871609; doi:10.3390/genes13020183)
Supplement: Supplementary file 1 [file genes-13-00183-s001.zip › Stover et al_GENES_COL1A1_Supp File S1_final.pdf]

Supplemental File S1: Characterization of chimpanzee COL1A1 exon 35 partial duplication

Separate Panel of Chimpanzees

A separate panel of chimpanzee samples was considered which included six induced pluripotent stem cell (**iPSC**) lines derived from chimpanzees (**Table S19**). These feeder-free iPSCs were previously reprogrammed and characterized. iPSCs were maintained and differentiated into mesenchymal stem cells (**MSCs**) and into osteogenic cells using previously described protocols [1].

Table S19: Separate Panel of Chimpanzee iPSC lines

Sample	Sex	Age (years)	Alternative ID
C1	M	16	C8861
C2	F	9	C3647
C3	M	9	C3649
C4	F	14	C40210
C5	F	17	C40280
C6	M	14	C3624

Testing for COL1A1 Exon 35 Partial Duplications in a Separate Panel of Chimpanzees

PCR was used to test for the presence of the COL1A1 exon 35 partial duplication in the panel of chimpanzees described above. Specifically, DNA was extracted from chimpanzee iPSCs (**Table S19**) using the ZR-Duet™ DNA/RNA MiniPrep (D7001, Zymo Research). DNA was quantified using the Qubit™ dsDNA HS Assay Kit (Q32851, Thermo Fisher Scientific) on a Qubit (Invitrogen). PCRs were run using the Q5® High-Fidelity 2X Master Mix (M0492S, New England Biolabs), 500nM of forward and reverse primers (**Table S20**), and 5ng of DNA. Primers were designed to amplify portions of COL1A1 regardless of the presence or absence of the exon 35 partial duplication (Set a1, Set a2, Set a3) or to amplify portions of COL1A1 only if the exon 35 partial duplication was present (Set b1, Set b2, Set b3). The PCR conditions included several steps: 1 cycle of 30 seconds at 98°C; 30 cycles of 10 seconds at 98°C, 30 seconds at a given anneal temperature (**Table S20**), and 10 seconds at 72°C; and 1 cycle of 2 minutes at 72°C. Non-template controls were included as negative controls, and chimpanzee samples with known COL1A1 exon 35 duplication profiles were included as additional controls (**Table S21**).

Amplified fragment lengths for each primer pair were determined by running the PCR products on a 1% agarose gel stained with ethidium bromide and using the Quick-Load® Purple 1 kb Plus DNA Ladder (N0550S, New England Biolabs) as a reference. Gels were visualized using a ChemiDoc MP Imaging System (Bio-Rad Laboratories).

Table S20: Primers for COL1A1 Exon 35 Partial Duplication Tests

Primer Set	Forward Primer Sequence (5' to 3')	Reverse Primer Sequence (5' to 3')	Amplicon (bp) [no duplication]	Amplicon (bp) [partial duplication]	Q5 Anneal Temp. (°C)
Set a1	CTGCTGGTCCCACTGGA	CCACGGTCTCCCTAGAAGAAA	205	205+329	67
Set a2	CCCATACTTGGCCCTTCC	GGGTCTTGGTACTCACAGG	207	331	66
Set a3	CTGCTGGTCCCACTGGA	GGGTCTTGGTACTCACAGG	264	388	66
Set b1	CCGTAAGTACAGAAGACCTGTTAAG	GGGTGAAGGCACAGCAG	0	212	65
Set b2	CCCATACTTGGCCCTTCC	GGGTGAAGGCACAGCAG	0	185	67
Set b3	CTGCTGGTCCCACTGGA	GGGTGAAGGCACAGCAG	0	242	68

Table S21: Controls for COL1A1 Exon 35 Partial Duplication Tests

Sample	Partial Duplicate of COL1A1 Exon 35
Pt71	unknown
Pt131	unknown
Pt349	unknown
Pt100	yes - homozygous
Pt102	yes - heterozygous
Pt103	yes - heterozygous

We found that two of the six iPSC samples tested are heterozygous for this partial duplication (**Table S22, Figure S13**). Specifically, primer Set a2 and primer Set a3 confirm that the positive control Pt100 is homozygous and that the positive controls Pt102 and Pt103 are heterozygous. They also reveal that C1 and C4 are likely heterozygous, while all other samples do not contain the exon duplication. Additionally, primer Set b1, primer Set b2, and primer Set b3 confirm that the positive controls Pt100, Pt102, and Pt103 have at least one copy of the duplicated exon. They also reveal that C1 and C4 have at least one copy of the duplicated exon, while all other samples do not have any copies of the duplicated exon. Primer Set a1 does not amplify as expected, so we excluded these results.

Table S22: Results of COL1A1 Exon 35 Partial Duplication Tests

Sample	Set a2 Results	Set a3 Results	Set b1 Results	Set b2 Results	Set b3 Results	Conclusion
Pt71	no duplication	no duplication	no duplication	no duplication	no duplication	no duplication
Pt131	no duplication	no duplication	no duplication	no duplication	no duplication	no duplication
Pt349	no duplication	no duplication	no duplication	no duplication	no duplication	no duplication
Pt100	homozygous	homozygous	at least 1 copy	at least 1 copy	at least 1 copy	homozygous
Pt102	heterozygous	heterozygous	at least 1 copy	at least 1 copy	at least 1 copy	heterozygous
Pt103	heterozygous	heterozygous	at least 1 copy	at least 1 copy	at least 1 copy	heterozygous
C1	heterozygous	heterozygous	at least 1 copy	at least 1 copy	at least 1 copy	heterozygous
C2	no duplication	no duplication	no duplication	no duplication	no duplication	no duplication
C3	no duplication	no duplication	no duplication	no duplication	no duplication	no duplication
C4	heterozygous	heterozygous	at least 1 copy	at least 1 copy	at least 1 copy	heterozygous
C5	no duplication	no duplication	no duplication	no duplication	no duplication	no duplication
C6	no duplication	no duplication	no duplication	no duplication	no duplication	no duplication

Figure S13: Results of COL1A1 Exon 35 Partial Duplication Tests

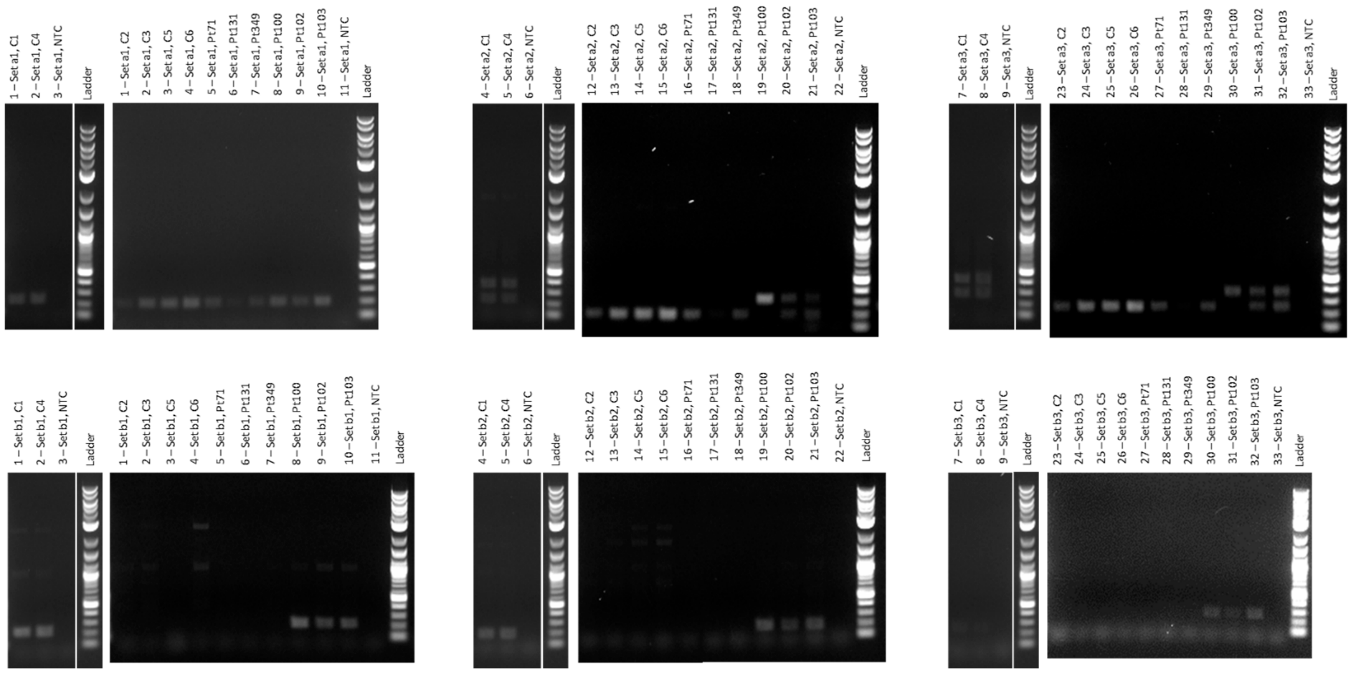

Examining RNA Expression in Relation to COL1A1 Exon 35 Partial Duplication

Quantitative real-time PCR (**qPCR**) was used to examine whether the COL1A1 exon 35 partial duplication is incorporated into the transcribed RNA molecule and whether its presence or absence affects RNA expression levels. Specifically, RNA was extracted from several chimpanzee iPSCs, iPSC-derived MSCs, and iPSC-derived osteogenic cells (**Table S19**) using the ZR-Duet™ DNA/RNA MiniPrep (D7001, Zymo Research) with a DNase (E1010, Zymo Research) treatment. RNA was quantified using the Qubit™ RNA BR Assay Kit (Q10210, Thermo Fisher Scientific) on a Qubit (Invitrogen). Complementary DNA (**cDNA**) was prepared using the Maxima FS cDNA kit for RT-PCR (K1642, Thermo Fisher Scientific). qPCRs were run on an Applied Biosystems QuantStudio 6 Flex Real-Time PCR System using the PowerUp™ SYBR™ Green Master Mix (A25742, Thermo Fisher Scientific), 400nM of forward and reverse primers (**Table S23**), and 5ng of cDNA. Two replicates of each sample were run in each qPCR. The qPCR conditions included several steps: 1 cycle of 2 minutes at 50°C; 1 cycle of 10 minutes at 95°C; and 40 cycles of 15 seconds at 95°C and 1 minute at 60°C. Melt curves were also determined.

The peak melt curve temperatures were compared across samples, and amplified fragment lengths for each primer pair were determined by running the qPCR products on a 1% agarose gel stained with ethidium bromide and using the Quick-Load® Purple 1 kb Plus DNA Ladder (N0550S, New England Biolabs) as a reference. Gels were visualized using a ChemiDoc MP Imaging System (Bio-Rad Laboratories). Additionally, expression level data were collected as Ct values, and these values were compared across samples.

Table S23: Primers for COL1A1 RNA Tests

Primer Set	Forward Primer Sequence (5' to 3')	Reverse Primer Sequence (5' to 3')	Amplicon (bp) [no duplication]	Amplicon (bp) [partial duplication]
Set c1	CTGCTGGTCCCACTGGA	ATCACCAGGTTCGCCTTTAG	128	165
Set c2	CTGCTGGTCCCACTGGA	TTTAGCACCAGCATCACCAG	140	177
Set a3	CTGCTGGTCCCACTGGA	GGGTCTTGGTACTCACAGG	~102	~138

From these RNA amplification results, we can see that the peak melt curve temperatures (**Figure S14**) and the amplified fragment lengths (**Figure S15**) are comparable across samples, suggesting that the partial duplication is not incorporated into the RNA molecule. In addition, within each cell type, expression levels are comparable across samples (**Figure S16**), indicating that the partial duplication does not affect gene expression levels.

Figure S14: qPCR Melt Curves

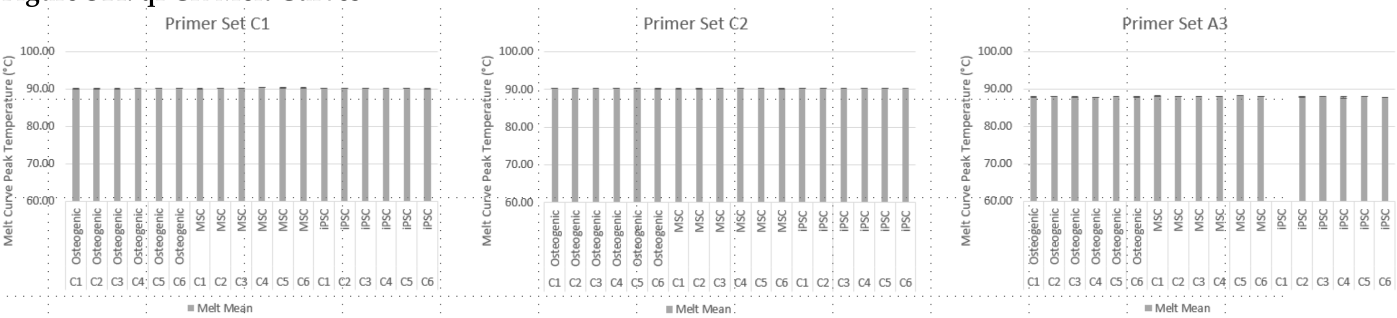

Figure S15: COL1A1 RNA Fragment Lengths

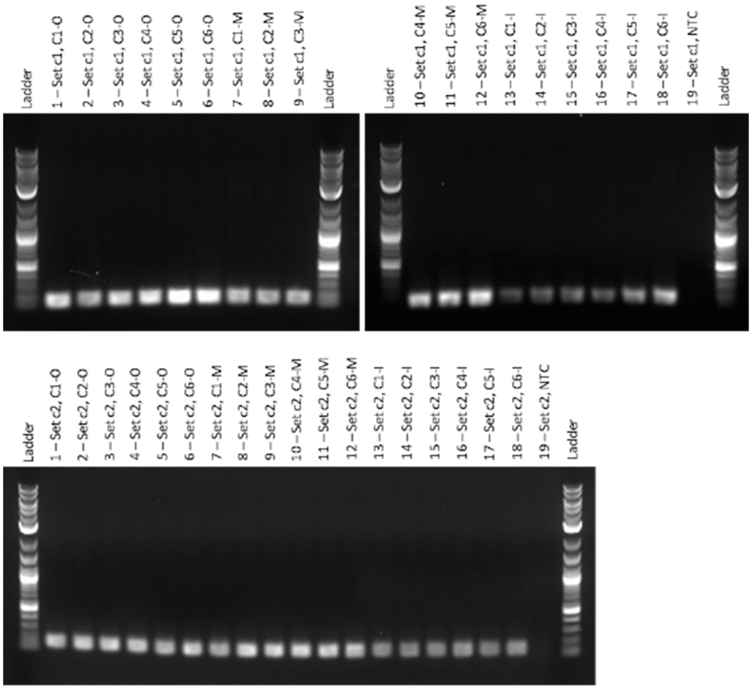

Figure S16: qPCR Expression Levels

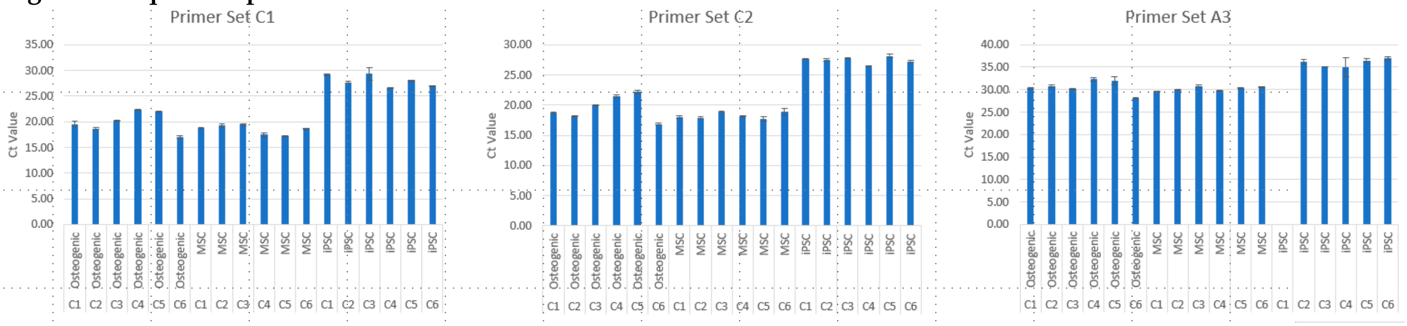

References

1. Housman G, Briscoe E, Gilad Y. Evolutionary insights into primate skeletal gene regulation using a comparative cell culture model. bioRxiv 2021.09.30.462680; doi:10.1101/2021.09.30.462680.
